# Enabling the Fabrication of Complex Soft Iontronics Using Multi‐Material 3D Extrusion Printing

**DOI:** 10.1002/advs.202505172

**Published:** 2025-09-24

**Authors:** Trevor J. Kalkus, Tamara V. Unterreiner, Målin Schmidt, Laura D. Wächter, Christina R. Schmitt, Ankit Mishra, Christine Selhuber‐Unkel

**Affiliations:** ^1^ Institute for Molecular Systems Engineering and Advanced Materials (IMSEAM) Heidelberg University 69120 Heidelberg Germany

**Keywords:** 3D printing, iontronics, ionic diode, mixed conductivity, soft robotics, strain‐stiffening

## Abstract

Iontronics can improve soft robotics, including wearable devices and environmental sensors, by replacing rigid electronics with viscoelastic materials that mimic biological tissue. Circuit components have been fabricated with soft materials that utilize ionic current, but the process can be tedious and widely applicable manufacturing methods are lacking, hindering the development of complex iontronic circuits for real‐world applications. With multi‐material 3D printing, this work demonstrates the ability to rapidly iterate ionic diode design and integrate these diodes within complex structures with biomimetic mechanical behavior. Print quality and material properties can be tuned by adjusting the concentration of the ink's components. To emphasize the rapid iteration enabled by 3D printing, a library of the ionic diodes with varying sensitivity to strain is evaluated. The utility of these ionic diodes is demonstrated by integrating them within logic circuits that respond to mechanical cues and demonstrate bio‐inspired strain‐stiffening behavior. These devices are functional directly from the 3D printer, are extremely flexible, and can be submerged in water without losing functionality. The adaptability afforded by multi‐material extrusion printing make it an ideal candidate for enabling the next generation of iontronics capable of advanced computational and mechanical functionality.

## Graphical abstract

Using multi‐material 3D printing, ionic diodes and logic circuits are created using only one fabrication technique and are integrated into metamaterial scaffolds with advanced mechanical behavior, paving the way for soft devices with tunable sensitivity to mechanical strain. This easily adaptable approach with high reproducibility creates a foundation for the accelerated progress in fields like soft robotics and wearable devices.

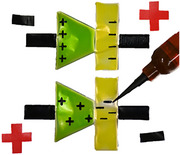

## 1 Introduction

Iontronics (or ionotronics) is an emerging field focused on advancing devices that use ions as charge carriers to generate current rather than electrons, promoting the utilization of non‐traditional materials, like hydrogels, to create viscoelastic conductive devices.^[^ ^1^, ^2^, ^3^, ^4^ ^]^ Because biology also uses ionic current for signaling and energy conversion, iontronics supports the development of bio‐inspired technology that matches the mechanical and electrical mechanisms found in nature.^[^ ^5^ ^]^ Whereas traditional electronics require rigid materials that do not interface ideally with soft environments, iontronics can be tuned according to their surroundings and purpose.^[^ ^6^, ^7^ ^]^ For example, soft robotics developed for environmental sensors can reduce potential damage to nature compared to plastic and metallic robotics.^[^ ^8^, ^9^ ^]^ Additionally, iontronics contribute to the development of wearable sensors,^[^ ^10^, ^11^, ^12^, ^13^ ^]^ drug delivery devices,^[^ ^14^ ^]^ self‐healing electronics,^[^ ^7^, ^15^ ^]^ energy harvesters,^[^ ^16^, ^17^, ^18^, ^19^ ^]^ ionic diodes,^[^ ^20^, ^21^, ^22^, ^23^, ^24^, ^25^, ^26^, ^27^, ^28^, ^29^ ^]^ ionic transistors,^[^ ^30^, ^31^, ^32^, ^33^ ^]^ and more. Despite this progress, the integration of these iontronic components within more complex circuits remains challenging due to fabrication limitations. As a nascent field, iontronics lacks a well‐established and widely applicable fabrication method.

The fabrication of an ionic diode, a basic component used to rectify current in logic circuits and beyond, requires the arrangement of several materials: an anion exchange material, a cation exchange material, an ionic reservoir, and encasing. In previous work, these materials are generally assembled by hand^[^ ^21^, ^22^, ^23^ ^]^ or within microfluidic channels.^[^ ^24^, ^25^, ^26^, ^27^, ^28^ ^]^ Although these approaches are suitable for simple demonstrations, these assembly methods become increasingly impractical as the circuit becomes more complex or if production needs to be upscaled. Furthermore, the electrodes that connect ionic diodes to the circuit are often composed of traditional metals,^[^ ^22^, ^23^ ^]^ which undermine the viscoelastic properties of the device. Electrodes composed of liquid metal^[^ ^34^, ^35^ ^]^ or aqueous salt reservoirs^[^ ^25^, ^26^, ^27^, ^36^ ^]^ can be utilized in soft circuits, but these provide design limitations and still do not contribute directly to the desired viscoelastic properties. Importantly, the combination of metallic or liquid components with viscoelastic components prevents the use of a single manufacturing method for the entire device. Due to these limitations, the established methods for iontronics hinder the development of complex soft circuits suitable for implantable and wearable devices.

**Figure 1:**
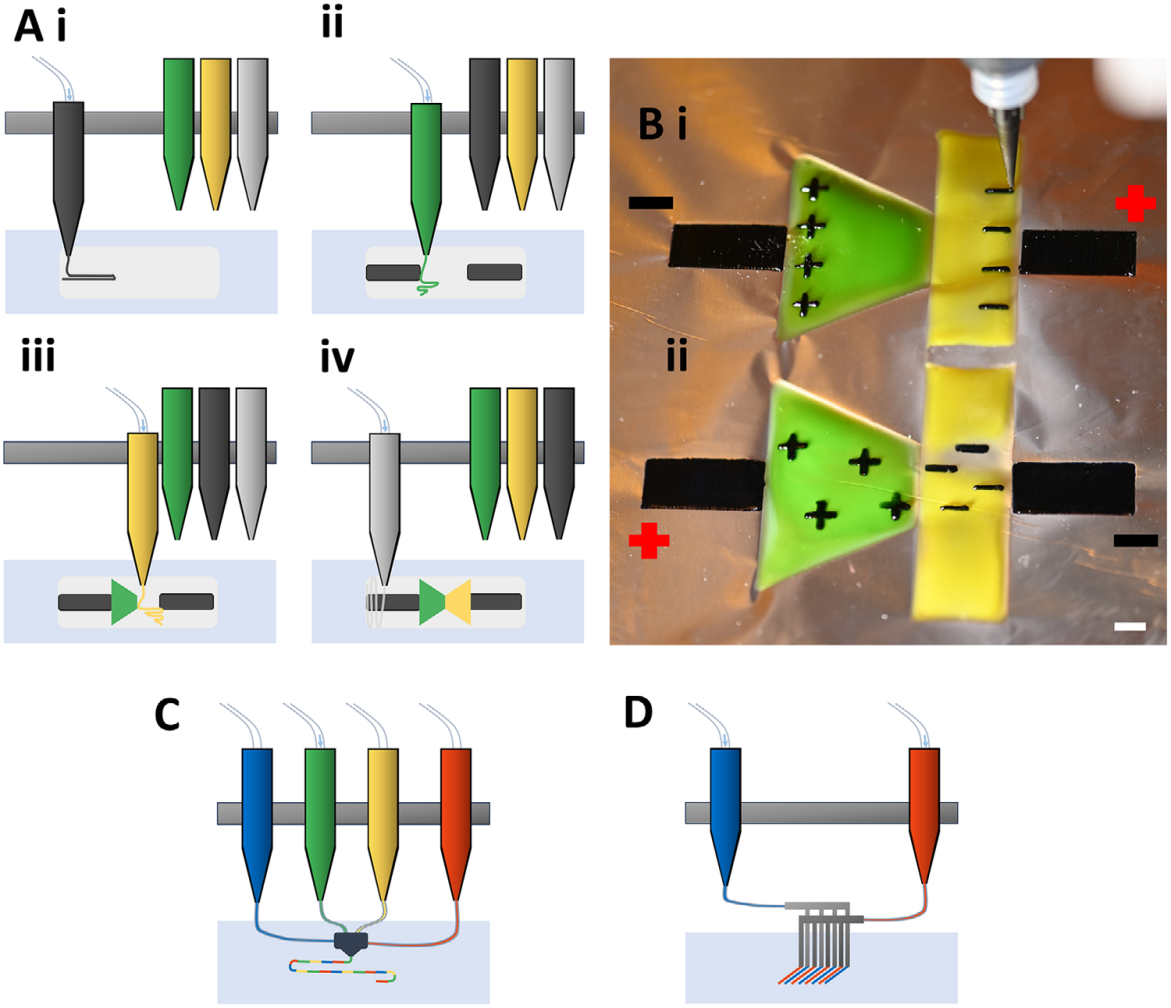
An overview of multi‐material extrusion printing and ionic diodes. A) The illustration shows the fabrication of an ionic diode using the multi‐material extrusion process demonstrated in this work. i) First, PDMS (shown in off‐white) is extrusion printed on the glass substrate. The mixed ionic‐electronic conducting hydrogel is being deposited and is shown in a dark color to represent the color of PEDOT:PSS. ii) The cation exchange hydrogel is printed followed by iii) the anion exchange hydrogel. These gels are represented by green and yellow respectively throughout this manuscript. iv) PDMS is printed again to encase the ionic diode. After every layer is deposited, the material is polymerized with UV light (not shown in this image). The entire process can be seen in Video S1 (Supporting Information). B) The nozzle of the extrusion printer can be seen after finishing the 3D printed representation of an ionic diode composed of multiple polyacrylamide hydrogels. The black hydrogel contains the conductive polymer mix PEDOT:PSS. The hydrogel dyed green (food dye) represents a hydrogel that selectively permits the diffusion of cations while limiting the diffusion of anions, whereas the hydrogel dyed yellow (food dye) represents a hydrogel that selectively permits the diffusion of anions while limiting the diffusion of cations. The mobile charges are shown as black (+) and (‐) within the respective hydrogels. i) When a negative potential is applied, the charge‐selective hydrogels limit the movement of ions, and a charge depletion occurs at the interface of these hydrogels, resulting is very limited current. ii) When a positive potential is applied, no ion depletion occurs, and current flows freely. The white scale bar indicates 5 mm. Alternate multi‐material extrusion printing techniques could be used to fabricate iontronics, like using C) a multi‐channel nozzle^[^ ^43^ ^]^ or D) a multi‐nozzle array.^[^ ^45^ ^]^

Because circuit components like ionic diodes require several materials, multi‐material 3D printing provides a suitable but heretofore unexplored method for fabricating iontronics. Extrusion 3D printing has recently disrupted many research and manufacturing fields due to its versatility, its potential for rapid iteration and personalization, and repeatability.^[^ ^37^, ^38^, ^39^ ^]^ Furthermore, extrusion printing is well suited for the fabrication viscoelastic materials, as evidenced by the growing relevance of bioprinting.^[^ ^40^, ^41^, ^42^ ^]^ Most commercially available bioprinters can hold several cartridges of ink and deposit each ink independently, as shown in **Figure** **1**A. Exciting progress in the field of multi‐material 3D printing includes the development of multi‐channel nozzles that print from several cartridges^[^ ^43^, ^44^ ^]^ (Figure 1C) and multi‐nozzle arrays that can print an entire multi‐material layer in a single pass^[^ ^44^, ^45^ ^]^ (Figure 1D). Any of these approaches are suitable for the rapid advancement of iontronics.

In this work, we fabricate ionic diodes and logic circuits using multi‐material extrusion 3D printing as the sole manufacturing method. The inks designed for printing the anion exchange hydrogel and the cation exchange hydrogel are related to previous work on iontronics and ionic diodes.^[^ ^17^, ^20^, ^21^, ^22^, ^23^, ^24^, ^25^, ^26^, ^27^, ^28^, ^29^, ^43^ ^]^ Where previous work used metals or ionic reservoirs, we instead embed a mixed ionic‐electronic polymer blend, poly(3,4‐ethylenedioxythiophene): polystyrene sulfonate (PEDOT:PSS), within a polyacrylamide hydrogel to engineer a material that can serve as an electrode and ionic reservoir for ionic diodes. One polymer (PEDOT) supports the transport of electrons, whereas the other (PSS) supports the transport of ions. Because many biological systems (neurons, muscle cells, etc.) naturally utilize ionic signaling but manmade technology mostly utilizes electronic signaling, this mixed ionic‐electronic conductor can bridge electronic and ionic systems.^[^ ^46^, ^47^, ^48^ ^]^ Literature provides examples of PEDOT:PSS hydrogels that have been 3D printed to interface with tissue or to act as a strain sensor.^[^ ^48^, ^49^, ^50^, ^51^, ^52^, ^53^ ^]^ However, mixed‐conductivity hydrogels have not yet been used in multi‐material printing to enable computational ionic circuits, like ionic logic gates.

To highlight the adaptability of multi‐material 3D printing for iontronics, we first demonstrate how material properties (printability, stiffness, and conductivity) can be tuned by adjusting the quantity of particular components in the ink (the rheological modifier, the crosslinker, and the conductive polymer). Because soft devices have wide‐ranging applications, from wearable devices to environmental sensors, the ability to adjust material properties according to the application underscores the appeal of 3D printing. We then emphasize the ability to rapidly iterate iontronic devices by 3D printing several new designs for ionic diodes that demonstrate different sensitivities to mechanical strain. The underlying mechanism of the ionic diode is outlined in the 3D printed representation shown in Figure 1B. Finally, we integrate ionic diodes within strain‐stiffening structures^[^ ^54^ ^]^ to create iontronic logic gates responsive to mechanical input. The resulting device exhibits both advanced electrical behavior and complex mechanical behavior, fully utilizing the advantages of viscoelastic iontronics. Using previously established fabrication methods, this iterative prototyping and integration of iontronic circuits would be tedious, labor‐intensive, and challenging to achieve, whereas we implemented changes by simply updating the 3D models for the extrusion printer.

## 2 Results and Discussion

**Figure 2:**
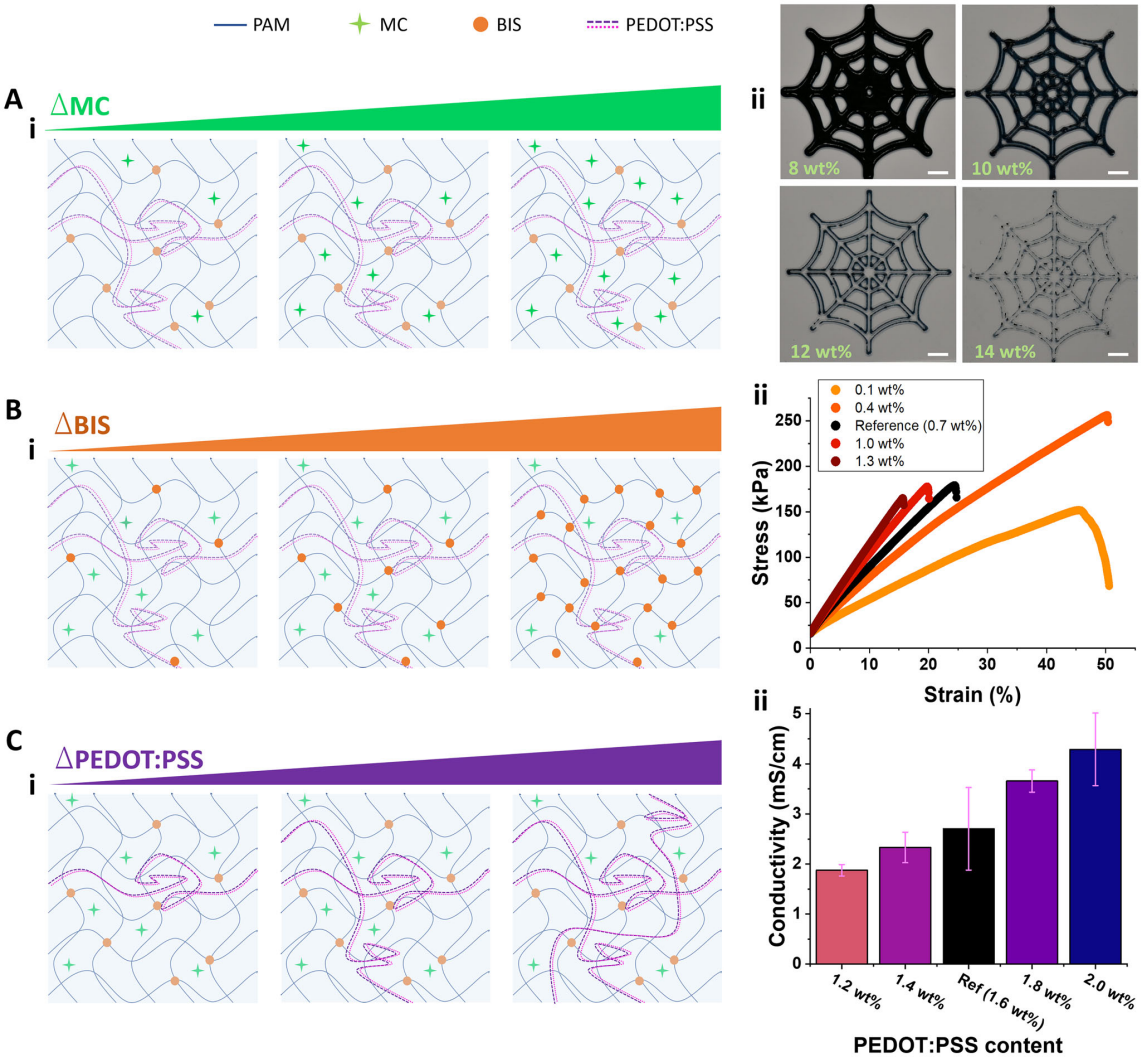
Tuning printing resolution, hydrogel stiffness, and hydrogel conductivity by adjusting ink composition. A i) An illustration of the polyacrylamide (PAM) hydrogel with increasing methylcellulose content (MC), the rheological modifier. ii) 3D printed dark web patterns show that increasing the MC content results in higher print resolution, but also that too much MC can limit the ink flow and reduce print continuity. The MC content is indicated with green text in the respective image. The scale bars indicate 5 mm. B i) An illustration of the PAM hydrogel with increasing crosslinker content (BIS). ii) Tensile tests reveal that an increase in BIS content results in stiffer hydrogels that break with less strain. These trials were performed in triplicate, and this figure shows single representative measurements of each BIS concentration to avoid overcrowding the plot. C i) An illustration of the PAM hydrogel with an increasing content of conductive polymer mix (PEDOT:PSS). ii) An increase in PEDOT:PSS content results in increasing conductivity due to the increase of charge carriers and conductive pathways. Measurements were taken in triplicate, and results are shown as the mean ± standard deviation.

One freedom offered by 3D printing soft materials is the ability to easily tune material properties according to the desired application. To demonstrate the optimization of the PEDOT:PSS ink, we evaluated the influence of three major components, the thickening agent or rheological modifier (methylcellulose, MC), the crosslinker (N,N′‐methylenebis(acrylamide), BIS), and the conductive polymer network (PEDOT:PSS), on printing behavior and the properties of the resulting hydrogel (**Figure** **2**). To analyze and compare the influence of each component, we first establish a reference ink composed of 12 wt.% MC, 0.7 wt.% BIS, and 1.6 wt.% PEDOT:PSS. We then create inks that vary the concentration of each component (MC, BIS, and PEDOT:PSS) independently and characterize those inks by assessing the ink's rheological properties, the ink's printability, the polymerized hydrogel's stiffness, and the hydrogel's conductivity. Rheological measurements of the inks (Section S1, Supporting Information) contribute to an ongoing effort to establish standardized characterization methods in the field of soft material extrusion printing.^[^ ^55^, ^56^, ^57^, ^58^ ^]^

While rheological analysis can help elucidate the flow characteristics of inks, it does not replace the practicality of directly evaluating the print quality, or printability, of the inks.^[^ ^55^ ^]^ Although printing parameters (including nozzle diameter, print speed, and extrusion pressure) can be altered to accommodate different inks and provide the best possible print,^[^ ^59^ ^]^ we printed each ink with the same parameters (0.41 mm nozzle inner diameter, 20 mm s^−1^ print speed, 100 kPa pressure) to enable the comparison between the different compositions, as seen in Figure 2A. The strand width of the 3D printed dark web facilitates the assessment of print resolution. Of the three components assessed, we found that MC content had the greatest impact on printing resolution. Increasing MC content results in increasing viscosity, which allows the print to remain stable after printing and retain a higher print resolution. Below a certain viscosity, the print will not hold its shape and spread outward, but above a certain viscosity, the extrusion nozzle may become clogged. We found that the PEDOT:PSS concentration also impacted printing resolution to a lesser extent, but that crosslinker (BIS) concentration had no meaningful impact on viscosity or printability (Sections S1 and S2, Supporting Information).

After the ink is extruded, UV irradiation is used to trigger free radical polymerization to form the hydrogel network, and mechanical properties were assessed using tensile characterization. The slope of the stress‐strain plot relates to the material's elastic modulus, the shape of the plot reveals the material's behavior under strain, and the material's maximum strain can be indicated by the breaking point.^[^ ^60^ ^]^ We 3D printed a band (10 mm width, 30 mm length, 0.6 mm thickness) of each hydrogel material to assess the tensile behavior as seen in Figure 2B. Of the three components assessed, crosslinker concentration had the greatest impact on the maximum strain that could be achieved before breaking. Relatedly, the lowest BIS concentration (0.1%) demonstrates the most gradual slope, stretching the farthest with the least amount of force. As maximal crosslinking is approached with the reference hydrogel (0.7 wt.% BIS), increased BIS content results in only minor changes to tensile behavior. On the other hand, if too little crosslinker is added, the hydrogel can be too soft to handle easily without breaking. We found that MC content had a relatively minor impact on tensile behavior, and that the impact of PEDOT:PSS on stiffness is dependent on the hydrogel fabrication method, which is examined in more detail in Section S4 (Supporting Information). 3D printing likely aligns PEDOT:PSS chains during the extrusion process, and these elongated polymers seem to contribute to increased stiffness compared to random polymer arrangements found in cast molded hydrogels. This effect has been previously observed with 3D printed PEDOT:PSS hydrogels.^[^ ^48^, ^61^ ^]^ This finding underscores that manufacturing methods directly impact material properties, and this must be considered when utilizing extrusion 3D printing and characterizing iontronic devices. Unlike PEDOT:PSS content, the BIS concentration has no meaningful impact on printability or material properties besides stiffness, and is thus the ideal component for fine‐tuning hydrogel stiffness.

We then evaluated the conductivity of 3D printed hydrogels with different compositions. The hydrogels presented in this work demonstrated conductivity values within the range of other 3D printed and tunable PEDOT:PSS hydrogels.^[^ ^46^, ^62^, ^63^, ^64^ ^]^ As expected, increasing the conductive polymer (PEDOT:PSS) content results in increased conductivity (Figure 2C). However, increasing MC content does negatively impact conductivity. Meanwhile, the crosslinker (BIS) content seems to have no impact on conductivity (Section S5, Supporting Information). This finding highlights another advantage of utilizing conductive polymers; hydrogels that use ionic charge carriers generally demonstrate reduced conductivity with increased crosslinker concentration because the diffusion paths of the ions become more convoluted.^[^ ^65^ ^]^ In contrast, the conductive pathways provided by PEDOT:PSS seem unaffected by crosslinking density at the concentrations investigated. Besides increasing PEDOT:PSS concentration, other potential methods for increasing the conductivity of PEDOT:PSS structures could include annealing with heat,^[^ ^48^ ^]^ exposure to acid, and the addition of ionic liquids.^[^ ^46^ ^]^

When considering the wide range of potential applications for soft iontronics, the three investigated components highlight the ability to optimize the fabrication process and material properties accordingly. Although increasing the PEDOT:PSS content increased conductivity, it also impacts every other material property, including the rheological behavior of the fluid ink, its printability, and the crosslinked hydrogel's stiffness. Fortunately, the MC content can be tuned to optimize fluid rheological properties and significantly alter the extrusion printing properties with minimal impact on the resulting hydrogel's stiffness. Offering even more adaptability, the BIS content can be tuned to optimize hydrogel stiffness with negligible impact on any other characteristic examined. Applying the insight gained from this thorough material characterization, we chose an ink composition that supported desired characteristics for the iontronic circuits presented in this work. Within the tested range, we minimized BIS content (0.1 wt.%) to allow for relatively high strains to be achieved without breaking, and minimized MC concentration (8 wt.%) while maximizing PEDOT:PSS concentration (2 wt.%) to maximize conductivity while still offering decent printing resolution. This ink was then used to print the ionic diodes and circuits presented.

**Figure 3:**
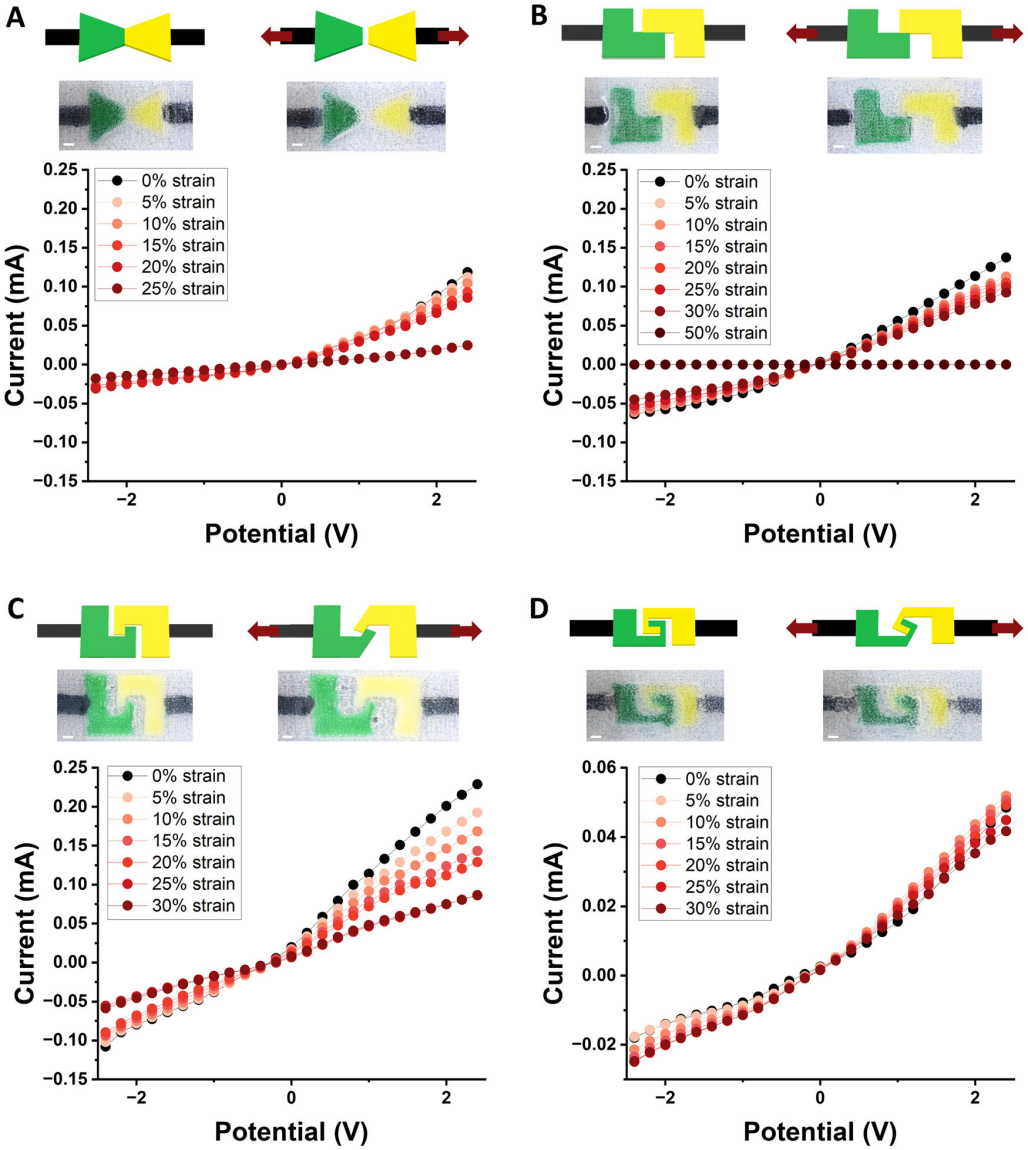
Iterative design of flexible ionic diodes with different strain sensitivity. Each panel characterizes a different ionic diode design with a current‐voltage (IV) plot to demonstrate the rectification behavior of the diode when experiencing a range of mechanical strain. Lines connecting points in all plots only serve to help guide the eye. Each line represents a single measurement of the same diode at the indicated strain. Diodes limit current in one direction when a negative potential is applied, while permitting higher currents in the opposite direction when a positive potential is applied. The two illustrated diagrams above each IV plot show the diode geometry associated with the plot before and during the application of strain. The red arrows indicate the direction of strain. The photos beneath these diagrams show printed material in the corresponding condition (before and during the application of strain). The green hydrogel represents the cation exchange hydrogel and the yellow hydrogel represents the anion exchange hydrogel. Food dyes were added to these gels for illustrative purposes. These gels were not used for the experimental measurements, nor do they perfectly represent the behavior of the materials discussed in this work. The white scale bar represents 2 mm. The different geometries are described as A) the traditional trapezoidal geometry, B) the parallel rectangle geometry, C) the hooked geometry, and D) the spiral geometry that is insensitive to strain.

To 3D print ionic diodes, we developed inks for printing a cation exchange hydrogel, which selectively allows the diffusion of cations while limiting the diffusion of anions, and an anion exchange hydrogel, which selectively allows the diffusion of anions while limiting the diffusion of cations. Previous work generally uses these materials in trapezoidal shapes to create ionic diodes, similar to the geometry shown in **Figure** **3**A.^[^ ^25^, ^26^ ^]^ With printable inks developed for these two hydrogel materials, the PEDOT:PSS gel as an electronic‐ionic interface, and an elastic polydimethylsiloxane (PDMS) ink to encase the hydrogels, we pioneered the use of multi‐material 3D printing for fabricating ionic diodes. The fabricated ionic diodes were functional directly from the printer with no need for additional functionalization or programming steps. The printing process can be viewed in Video S1 (Supporting Information).

Because a primary motivation for soft electronics is to utilize their ability to bend, deform, and stretch, the behavior of ionic diodes under strain is an important consideration. As seen in previous work,^[^ ^23^ ^]^ the resistance of the trapezoidal diode increases with strain. In our case, the interface between the two selective hydrogels became increasingly tenuous with increasing strain until they were separated at 25% strain. With the rapid prototyping capabilities afforded by multi‐material 3D printing, we iterated the diode design to explore how the diode geometry can change its response to strain. Rather than the trapezoidal shape, Lee et al. used side‐by‐side right triangles to design a highly flexible diode sensitive to strain.^[^ ^24^ ^]^ Similarly, we printed a diode composed of two parallel rectangles that could easily slide past each other when stretched, staying in contact but reducing the contact area with increasing strain, as seen in Figure 3B. As expected, this resulted in increased resistance with strain, and the components of the diode did not separate until a 50% strain was reached, increasing the range compared to the trapezoid geometry. While strain was only applied in a direction parallel to the circuit, we expect that the selective hydrogels would separate quickly if strain was applied in the perpendicular direction. This provides exciting opportunities for designing devices that are more sensitive to strain in one direction than the other. When implementing these first two diode designs, the user should desire a circuit that is sensitive to strain, resulting in a mechano‐responsive circuit. However, other situations might require a diode that retains its functionality without being impacted by stretching, in which case different geometries must be implemented.

We first tested a hooked design where the two selective materials would be pulled into each other instead of apart from each other with strain in the direction of the circuit (Figure 3C). However, because of the flexibility of the components, the materials were still able to bend and slide past each other. In fact, this iteration demonstrated a stronger response to strain than the parallel rectangles specifically designed for strain sensitivity. The increase in resistivity in response to strain is related to the width of the selective hydrogels at their interface because resistance is related to the cross‐sectional area of the circuit. When the hook experienced strain, longer portions of the thin geometry became the main conduit of current, dramatically increasing resistance. Comparatively, the wide geometry of the parallel rectangles allowed current to flow relatively easily even when the strain reduced the contact area between the selective gels.

An ionic diode that is insensitive to strain was achieved by designing the selective hydrogels in a spiral around each other (Figure 3D). As this geometry experiences strain, the contact area between the selective hydrogels remains the same despite the deformation that occurs. This diode behaves largely the same under no strain as it does with high strain. However, due to the thin width of the design, this diode results in much more resistance than the other designs, and further iteration and exploration would likely result in improved performance. Diode iterations can be printed rapidly in sequence or even simultaneously; updating the 3D printing model is the only change necessary for rapid iteration. Hence, the use of multi‐material 3D printing unlocks prototyping capabilities previously inaccessible to soft iontronics. Although we show 3D models with hard angles, organic shapes can also be printed like the yin‐yang diode demonstrated in Figure S9 (Supporting Information). These materials can also be printed onto various substrates. Previous work created flexible diodes using extremely flexible VHB tape,^[^ ^23^, ^24^ ^]^ and we were also able to print on VHB tape and show that the diode still performs even when twisted, folded, and submerged in water (Figure S9, Supporting Information). Notably, because the conductive polymer network is interpenetrated within the hydrogel, the ionic reservoir does not diffuse out of the iontronic device even when submerged in distilled water, although hydrogel swelling will impact ion flow^[^ ^66^, ^67^, ^68^ ^]^ (Figure S7, Supporting Information). All four diode designs highlighted in Figure 3 returned to their original shape and original performance after experiencing strain and remained stable through several cycles of strain.

**Figure 4:**
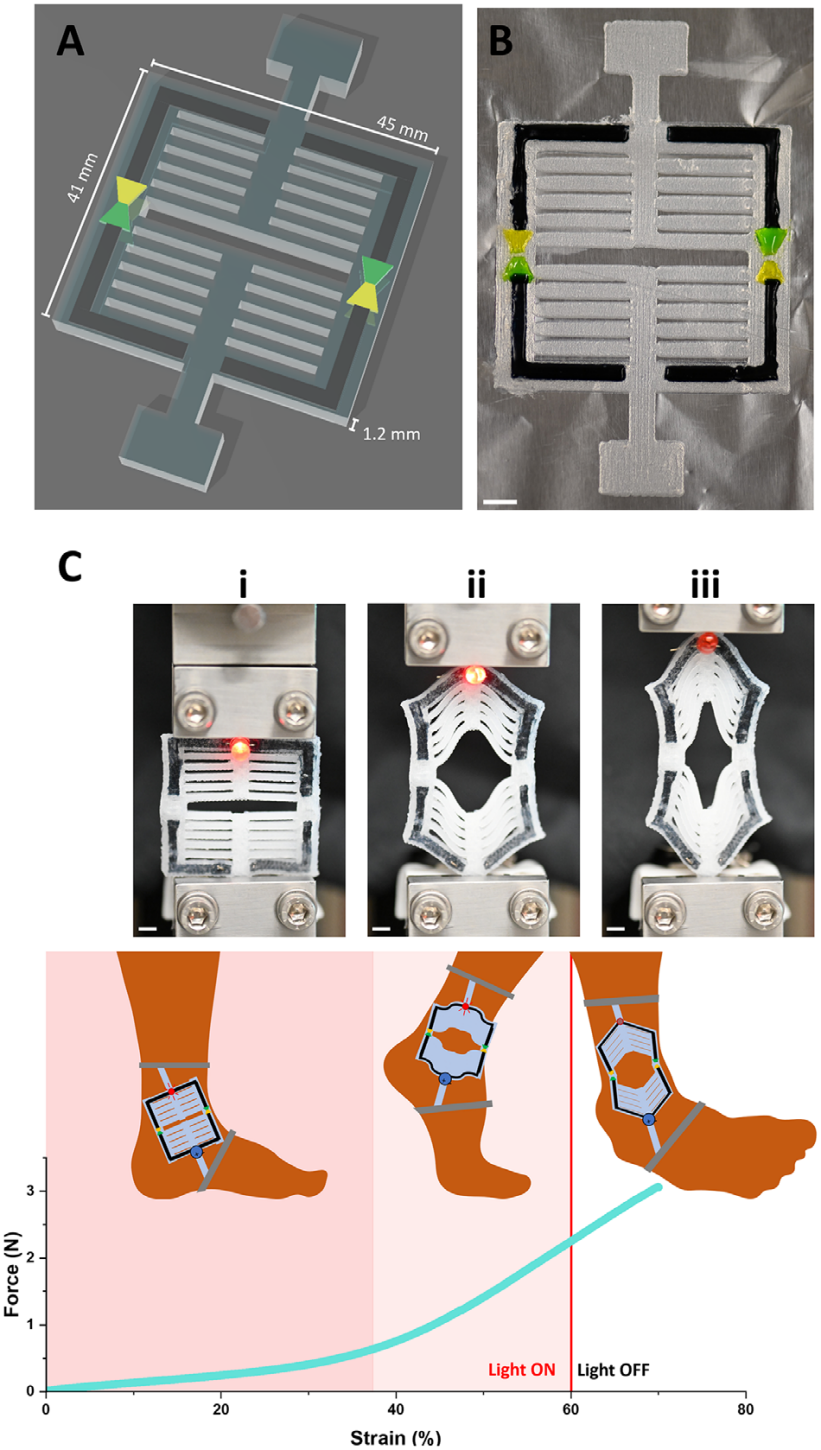
Iontronics that use ionic diodes are incorporated into a 3D strain‐stiffening device using multi‐material 3D printing. A) A 3D model of the strain‐stiffening device and the incorporated ionic diodes. The different materials are indicated by different colors: PDMS is translucent, the PEDOT:PSS functionalized hydrogel is black, the cation exchange hydrogel is green, and anion exchange hydrogel is yellow. B) An image of the iontronics being incorporated in the strain‐stiffening device during the printing process. The selective hydrogels are dyed to match colors in Panel A, and this photographed structure is used only for demonstration, not for experimental measurements. The white scale bar indicates 5 mm. C) Images and illustrations aligned with a force‐strain curve to demonstrate a potential use case for the extrusion printed electronics in a wearable device. i) At low strain, the device is very flexible and can be extended with little force, allowing the joint to move freely. The electronic circuit powers a light indicating normal activity. ii) The steepening slope at the critical strain ≈38% highlights the strain‐stiffening behavior of the 3D printed design. With moderate strain, the device stiffens to help prevent overextension and injury. The LED remains on and unaffected. iii) When the joint is overextended and causes extreme strain, the electronic circuit opens and the light loses power to indicate the risk of injury. The plot shows a single measurement. The process is fully reversible within the elastic regime of the device and the circuit returns to its previous state as strain is reduced as seen in Video S2 (Supporting Information) and especially in Video S3 (Supporting Information), where the force‐strain plot is shown in real time and through three cycles of strain. The white scale bar indicates 5 mm.

Along with the rapid exploration of diode design, multi‐material extrusion printing also allows these diodes to be incorporated directly and seamlessly into circuits and structures suitable for real‐world applications. Skin and other soft biological tissues exemplify strain‐stiffening behavior; they stretch with little resistance until a certain threshold, a critical strain, at which point they provide more resistance to strain.^[^ ^54^ ^]^ Such material behavior can be desired in wearable devices, like joint braces that help prevent injury, and our lab has previously published a bio‐inspired design that has tunable strain‐stiffening behavior.^[^ ^54^ ^]^ In **Figure** **4**, we demonstrate that ionic diodes can bestow addition functionality to an iontronic circuit incorporated into this strain‐stiffening structure. We opted to utilize the traditional trapezoidal diode geometry to fabricate a circuit that will indicate when a specific strain is surpassed. In wearable devices, this can identify movements that present a high risk for injury. In this example, we can power a LED that has been incorporated into the extrusion printed strain‐stiffening circuit. The LED remains powered after the stiffening behavior begins, but loses power at the strain where the diode components become separated. This process is entirely reversible, and the LED becomes illuminated again when the strain is reduced (Videos S2 and S3, Supporting Information). While most soft electronics focus on the bulk properties of their materials, this demonstration highlights how 3D printing makes functional geometries easily accessible. Like the iterative design of the ionic diodes, this strain‐stiffening structure can be adjusted, or personalized, according to the user. This demonstration breaks ground on fabricating iontronic devices with electrical computational capability as well as advanced mechanical behavior, a feat made accessible by the multi‐material extrusion printing approach.

**Figure 5:**
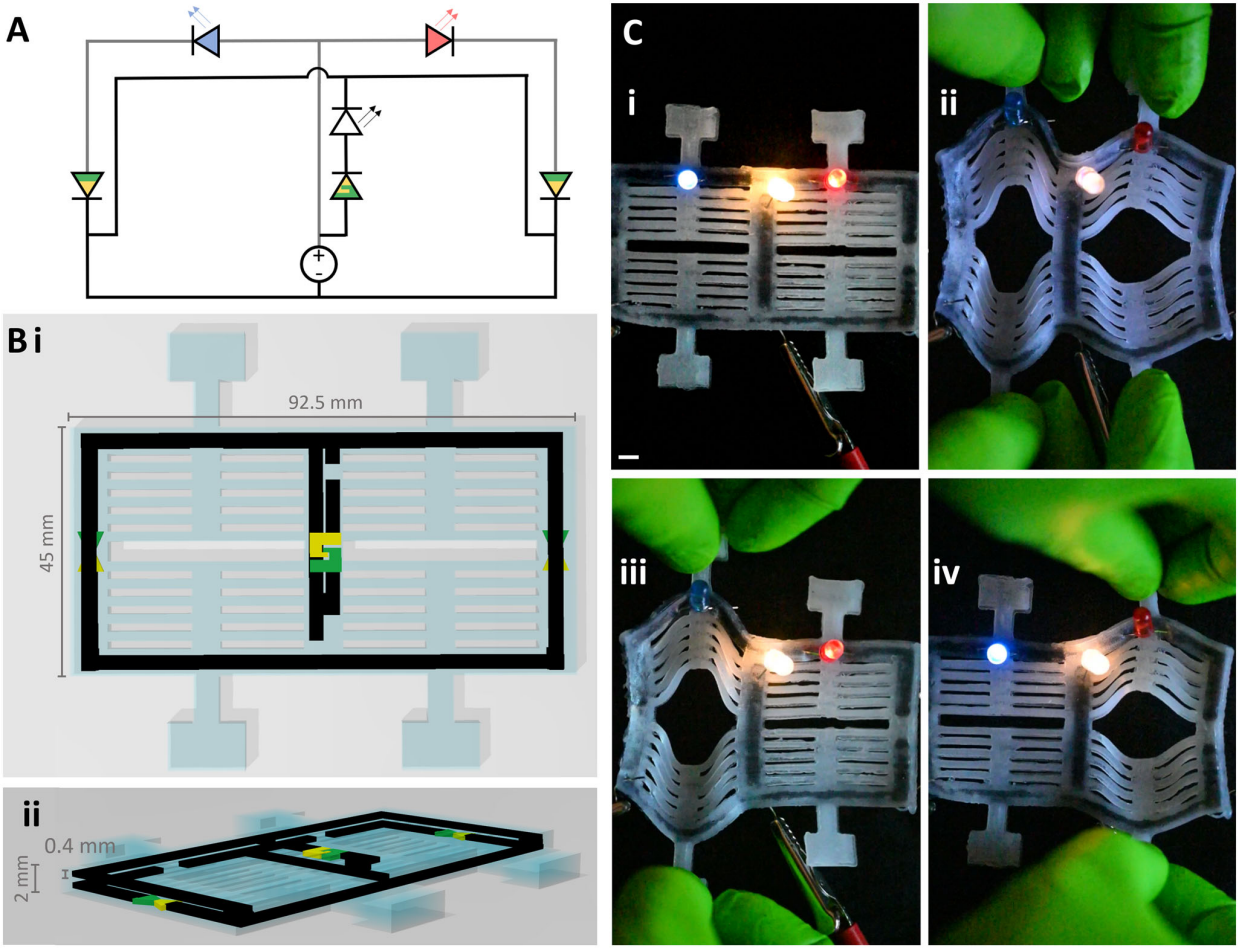
3D printed logic circuit responsive to mechanical cues. A) A diagram of the ionic circuit. The gray lines indicate the parallel circuit that is printed at a different height than the black lines. LEDs are indicated by color with arrows representing the light emitted. Ionic diodes are green and yellow to match the color scheme used in this work. The ionic diodes that are half green and half yellow indicate the traditional trapezoid geometry and will break the circuit when stretched. The ionic diode with the green and yellow hooked together indicate the hooked diode geometry and does not break the circuit when strained. B i) The 3D model of the entire device from above. ii) The 3D model of the entire device from an angle, allowing a perspective of the stacked circuits. C i) With no mechanical input, all LEDs remain lit. ii) When both left and right sides are stretched, both the blue and red LEDs are disconnected. The white LED remains lit, indicating that power is provided and the parallel circuit remains unbroken. iii) When the left side is stretched, the blue LED is disconnected while the white and red LED remain illuminated. iv) When the right side is stretched, the red LED is disconnected while the white and blue LED remain illuminated. The white scale bar indicates 5 mm.

Building on this, we can create complex logic circuits that are responsive to mechanical input. Diodes are the only component needed to make AND/OR logic gates, and ionic diodes have been implemented to create ionic logic circuits.^[^ ^23^, ^24^, ^25^, ^26^ ^]^ Not only can the diode be used to rectify current, the strain‐sensitive diodes can essentially be an on/off switch for the input current if the mechanical strain causes a break between the diode components. The computational complexity made accessible by multi‐material 3D printing is thus far unprecedented among entirely viscoelastic iontronic devices. To begin to scratch the surface of the potential of this approach, we created a simple logic circuit with two mechanical inputs and three LEDs that can describe the state of the circuit (**Figure** **5**; Video S4, Supporting Information). One white LED is always on to indicate that power is being provided, a blue LED turns off when mechanical strain is applied on the left side of the circuit, and a red LED turns off when mechanical strain is applied to the right side of the circuit. To power the white LED while the others are off required the use of a parallel circuit stacked in the z‐dimension above the other circuit. While the diodes and circuit shown in this work previously were planar, this circuit takes advantage of the 3D aspect of the fabrication method. Two diodes with the traditional trapezoid shape are used in series with the blue and red LEDS to break the circuit with mechanical strain. A hooked diode was used in series with the white LED to emphasize that this geometry can maintain its connection when the trapezoid geometry does not. This demonstration acts as a predecessor to much more complex circuits that can be facilitated by multi‐material 3D printing. Devices could easily be designed to also account for strain in the x‐ and z‐dimensions, and more complex components, like ionic transistors,^[^ ^30^, ^31^, ^32^, ^33^ ^]^ could be incorporated.

## 3 Conclusion

To attest to the potential of multi‐material 3D printing for rapidly advancing iontronics, we used a single fabrication process to create complex devices with advanced mechanical behavior capable of utilizing ionic current for computational functions. This approach enabled a level of complexity and adaptability heretofore unseen for iontronics. The demonstrated iontronic devices are functional directly from the printer without need for further functionalization, and can be twisted, folded, and submerged in water without losing functionality. Neither the computational behavior nor the mechanical behavior represents the bulk properties of the material used. Instead, the computational behavior arises from the interaction between different materials and is accomplished by multi‐material printing, and the mechanical behavior arises from the printed metamaterial geometry. We also highlight that these properties can be tuned and personalized by optimizing the ink composition or the device geometry. Based on the geometry of an ionic diode, a device can produce a digital, an analog, or no signal in response to strain. Multi‐material printing makes prototyping, personalization, complex circuit design, and integration within soft devices accessible for iontronics in a highly reproduceable manner, lowering the barrier to the next generation of soft technologies. In this work, inks are extruded directly onto an unfunctionalized glass substrate. Future work could consider alternative approaches like liquid‐in‐liquid extrusion printing techniques that offer advantages like the ability to print overhanging structures^[^ ^62^ ^]^ and to print filaments with a diameter of 1 µm^[^ ^69^ ^]^ but would require very different rheological considerations. Compared to previously established methods for fabricating iontronics, we reduce the required time, effort, and wasted material. By applying multi‐material 3D printing to the fabrication of iontronics, we provide a practical foundation for developing devices for real‐world applications, like wearable technology and soft robotics.

## 4 Experimental Section

### Materials

Acrylamide (AM), poly (3,4‐ethylenethioxythiophene):polystyrene sulfonate (PEDOT:PSS), methylcellulose (viscosity: 15 cP) (MC), N,N’‐methylenebisacrylamide (BIS), lithium phenyl(2,4,6‐trimethylbenzoyl) phosphinate (LAP), (3‐glycidyloxypropyl)trimethoxysilane (GOPS), hexacyanoferrate (II), hexacyanoferrate (III), potassium chloride, 2‐acrylamido‐2‐methyl‐1‐propanesulfonic acid (AMPS), (3‐acrylamidopropyl)‐trimethylammonium chloride solution (APTAC, 75 wt.% in water), 4‐methoxyphenol (MeHQ), tetrahydrofuran, fumed silica, 2‐hydroxy‐4’‐(2‐hydroxyethoxy)‐2‐methylpropiophenone (irgacure‐2949), hydroxy‐terminated poly(dimethylsiloxane) (PDMS), 3‐(trimethoxysilyl)propyl methacrylate, and dibutyltin dilaurate were purchased from Merck KGaA. Deionized water was sourced from a Milli‐Q EQ 7000. A photoinitiator stock solution was prepared by dissolving 340 mg LAP in 10 mL deionized water. A polymerization inhibitor 10x stock solution was prepared by dissolving 12.4 mg of MeHQ in 1 mL of deionized water, which was then diluted 10‐fold before use.

### Preparation of Inks for Printing Conductive Hydrogels

The primary hydrogel network is composed of polyacrylamide (PAM). Although PAM hydrogels are commonly used and easily tunable,^[^ ^70^, ^71^ ^]^ the low viscosity of the hydrogel precursor may be a reason it is only infrequently used for extrusion printing. The addition of PEDOT:PSS, as shown in this work, dramatically increases the ink viscosity and provides improved characteristics for extrusion printing. The addition of GOPS crosslinks PSS chains, creating a polymer double‐network within the hydrogel, insuring that PEDOT:PSS does not diffuse out of the gel or lose its structure.^[^ ^72^, ^73^ ^]^ When preparing the inks, PEDOT:PSS, AM, GOPS, and BIS were added to deionized water first, mixed, and sonicated for 1 min. The solution was then stirred continuously for 1.5–2 h to allow the PEDOT:PSS to become homogenously mixed. Then the LAP photoinitiator stock solution was added while stirring. The MC was added last and, due to the resulting viscosity, needed to be mixed with a spatula. Methylcellulose was chosen as the rheological modifier because it is commonly used in water‐based bioinks,^[^ ^74^ ^]^ and other options, like cellulose nanocrystals^[^ ^75^ ^]^ or hydroxyethyl cellulose,^[^ ^43^ ^]^ could be investigated in future iterations. Each of these inks contained 1.4 g AM and 0.4 mL LAP stock solution per 5 mL of distilled water. The PEDOT:PSS, MC, and BIS concentrations varied. For example, the reference ink contained 1.6 wt.% PEDOT:PSS, 12 wt.% MC, and 0.7 wt.% BIS which equated to 129 mg PEDOT:PSS, 970 mg MC, and 56 mg BIS per 5 mL of distilled water. The various PEDOT:PSS concentrations, 1.2, 1.4, 1.8, and 2.0 wt.%, respectively equated to 97, 113, 145, and 162 mg of PEDOT:PSS per 5 mL of distilled water, while all other components remained the same as the reference ink. The various MC concentrations, 8, 10, 14, and 16 wt.%, respectively equated to 620, 790, 1155, and 1350 mg MC per 5 mL of distilled water, while all other components remained the same as the reference ink. The various BIS concentrations, 0.1, 0.4, 1.0, and 1.3 wt.%, respectively equated to 8, 32, 81, and 106 mg BIS per 5 mL of distilled water, while all other components remained the same as the reference ink. After the inks were mixed, they were transferred to an extrusion printing cartridge, which was closed with a cap on the nozzle end and a plunger on the open end, and centrifuged for 10 min at 1400 RPM using a Heraeus Megafuge 8 centrifuge (Thermo Scientific) to remove air and settle the ink at the nozzle end of the cartridge.

Based on the exploration of parameters above, the ink formulation used in the diodes and strain‐stiffening devices was chosen to maximize both flexibility and conductivity. To do so, the lowest quantity of MC and BIS (8 and 0.1 wt.% respectively) and the highest quantity of PEDOT:PSS (2 wt.%) within the range tested were used. All other components remained the same as the reference ink.

### Preparation of Inks for Printing Ion Exchange Hydrogels

A batch of the ink for the hydrogel with selective transport of anions consisted of 700 mg AM, 200 mg BIS, 3 mL deionized water, 100 uL of MeHQ polymerization inhibitor stock solution, 50 µL of LAP photoinitiator stock solution, 525 mg MC, and 2 mL of APTAC. The ingredients were added in the order listed while stirring. The MC was allowed to completely dissolve by stirring for at least 15 min before adding APTAC, which was then stirred in gently with a spatula.

A batch of the ink for the hydrogel with selective transport of cations consisted of 500 mg AM, 200 mg BIS, 2 mL deionized water, 300 uL MeHQ polymerization inhibitor stock solution, 50 µL of LAP photoinitiator stock solution, 2.5 g AMPS, and 500 mg MC. The ingredients were added in the order listed while stirring, and needed to be stirred with a spatula after the addition of MC due to viscosity.

For both ion‐selective inks, a high crosslinker concentration was used to mitigate swelling when the hydrogel is submerged. The inhibitor mitigates polymerization in the extrusion nozzle to avoid clogging.

### PDMS Ink Preparation

5 g of hydroxy‐terminated PDMS was mixed with 600 µL of 3‐(trimethosysilyl)propyl methacrylate. Tetrahydrofuran (THF) was added to lower the viscosity and allow these components to mix. Then, 60 µL of the catalyst, dibutyltin dilaurate, was added. The mixture was stirred at 60 °C for 1.5 h until the THF was mostly evaporated. Separately, 50 mg of the photoinitiator, irgacure‐2949, was dissolved in 50 mL of THF and then added to the mixture. This was stirred without heating for 1 h or until the THF was mostly evaporated again. This protocol was developed based on a previously published photocurable PDMS recipe.^[^ ^76^ ^]^ Fumed silica (750 mg) was added as a rheological modifier to provide the desired viscosity and flow characteristics. It is found that fumed silica worked well as rheological modifier in this PDMS ink but that it did not result in the desired rheological properties when used in the water‐based inks. Similarly, methylcellulose dissolves better in aqueous environments and was therefore not suitable as a rheological modifier in the PDMS ink.

### Rheology

Rheological measurements were conducted with a TA Instruments HR20 rheometer equipped with an 8 mm stainless steel plate geometry. The geometry gap was set to 500 µm and a light beam platform was used to allow for UV polymerization. The ink filled the geometry gap, and excess ink was removed. To limit drying during the measurements, a wet Kimtech wipe placed around the sample within the solvent trap aided in providing a humid environment. The recovery behavior of the hydrogel was measured first using three sequential steps: a low shear rate of 0.01 s^−1^ to simulate nearly no stress for 200 s, followed by an increased shear rate of 1000 s^−1^ to simulate printing for 100 s, and finally a return to the initial shear rate of 0.01 s^−1^ for 300 s. Shear‐thinning behavior was evaluated next by increasing the shear rate logarithmically from 0.01 to 1000 s^−1^. A 35‐s recovery at a shear rate of 0.01 s^−1^ was introduced after the shear thinning step to allow the sample to stabilize before the subsequent flow initiation step. The flow initiation behavior was measured by logarithmically increasing the stress from 10^−4^ to 1 kPa. Measuring these characteristics using this sequence did not alter the results compared to measuring them independently, as shown in Figure S2 (Supporting Information).

After the characteristics of the unpolymerized ink were measured, UV light was used to cure the ink and crosslink the hydrogel in the geometry gap. The UV lamp intensity was set at 22.4 mW cm^−^^2^. Crosslinking was monitored by measuring the loss modulus and storage modulus using a constant angular frequency of 6.3 rad s^−1^ for 1 min. The UV illumination commenced 18 s into the measurement to initiate the curing process and lasted for 60 s. After the curing process, the storage and loss moduli were measured logarithmically while increasing the angular frequencies from 0.1 to 100 Hz to measure the stiffness and stability of the hydrogel.

### Extrusion Printing

Preliminary trials were performed using an Inkredible+ bioprinter (Cellink, Sweden). Otherwise, a BioScaffolder 5.3/CB (GeSiM, Germany) was used for extrusion printing. Inks were loaded into amber cartridges (to limit light exposure) with 10 mL capacity. All printing was done using pneumatic pressure for extrusion. The bioprinter configuration had two holders for pneumatic printing, so inks were manually exchanged during multi‐material prints, like the diodes integrated into the strain‐Mstiffening structure. The inks were printed onto 50 mm by 50 mm glass slides with no special treatment or functionalization. Separate stl files were designed for each material. The 3D models were made using Microsoft 3D Builder. Most prints were performed using a nozzle with 0.41 mm inner diameter, a pressure of 100 kPa, and a printing speed of 20 mm s^−1^. The printing direction of the infill lines was rotated 90° every layer, creating a perpendicular pattern with the layers above and below. The layer height was set to 0.2 mm, and typically each material was applied in two layers with perpendicular print directions. For example, printing a diode would first involve printing two layers (0.4 mm total) of PDMS, then two layers of each of the four inks in their respective patterns (0.4 mm height total), and then two more layers of PDMS, resulting in a soft, ionic diode with a 1.2 mm thickness.

### Tensile Tests

For each ink, a strip ≈40 mm long, 10 mm wide, and 0.6 mm thick was tested immediately after being printed to mitigate changes that might occur from dehydration. The strip was clamped into the ZwickRoell (Germany) tensile testing machine with 30 mm distance between the clamps and a 100 N sensor. An initial load of 0.1 N was reached before the measurement began. The strip was stretched at a rate of 50 mm min^−1^ and stopped after the material broke. This was repeated at least three times with each material. The diodes were also tested with a clamping distance of 30 mm, but they were stretched incrementally. At each increment, a voltage sweep was performed at a rate of 0.2 V every 0.1 s from −2.6 to 2.6 V, and Ag/AgCl electrodes were used to interface with the PEDOT:PSS hydrogel. For the strain‐stiffening device, a cyclic program was used that travelled at a rate of 50 mm min^−1^ and reached a maximum of 70% strain.

### Conductivity and Electrical Characterization

Samples with an approximate length of 10 mm, width of 4 mm, and height of 0.6 mm were extrusion printed. These dimensions were measured for each print to calculate conductivity. Extrusion printing parameters were changed with each ink to attempt to keep the printing results as similar as possible. For example, higher printing pressure was used with more viscous inks so that gaps would not be left in the lattice that was printed. An electrolyte solution was mixed using 100 mM hexacyanoferrate (II), 100 mM hexacyanoferrate (III), and 1 M KCl. Hexacyanoferrate has a very low redox potential, making it an ideal candidate for measuring ionic conductivity.^[^ ^17^ ^]^ This solution was absorbed into a graphite felt and a platinum wire was used as a current collector to connect to a Keithley 2450 source meter (Tektronix, USA) for conductivity characterization. To ensure that no resistance was contributed by the electrodes themselves, the resistance was measured at two different lengths (8 and 4 mm) and subtracted to get the resistance of a 4 mm length of hydrogel. In this process, it is found the resistance contributed by the electrodes was negligible.

### Statistical Analysis

Where applicable, data is presented as the mean value ± the standard deviation. The sample size for each measurement was *n* = 3 unless stated otherwise. Data was plotted using OriginLab software.

## Conflict of Interest

Heidelberg University holds patents: EP3801404A1, EP3445415A1 (Selhuber‐Unkel is involved).

## Author Contributions

T.J.K. and C.S.U. conceived the project. T.J.K. and T.V.U. designed the experiments. T.J.K., T.V.U., L.D.W., and C.R.S. collected the data. M.S. provided laboratory training, guidance, supervision, and imaging, and aided in developing the strain‐stiffening design. A.M. provided helpful chemistry insights, supported conductivity characterization, and ink formulation suggestions. T.J.K. drafted the writing, M.S. helped design figures, and all authors contributed to the final manuscript.

## Supporting information



Supporting Information

Supplemental Video 1

Supplemental Video 2

Supplemental Video 3

Supplemental Video 4

## Data Availability

The data that support the findings of this study are available from the corresponding author upon reasonable request.
