# Supplementary material for: Enabling the Fabrication of Complex Soft Iontronics Using Multi‐Material 3D Extrusion Printing
Source: Adv Sci (Weinh). 2025 Sep 24;12(42):e05172. doi: 10.1002/advs.202505172 (PMC12622534; doi:10.1002/advs.202505172)
Supplement: Supplementary file 1 — Supporting Information [file ADVS-12-e05172-s001.pdf]

Supporting Information

Enabling the fabrication of complex soft iontronics using multi-material 3D extrusion printing

Trevor J. Kalkus*, Tamara V. Unterreiner, Målin Schmidt, Laura D. Wächter, Christina R. Schmitt, Ankit Mishra, Christine Selhuber-Unkel*

Supporting characterization of the inks and hydrogels are presented in the order of fabrication. Beginning with rheological characterization of liquid inks to help create more standardized understanding of extrusion printed materials, we then present data characterizing the crosslinking process which results in hydrogels that are then characterized for conductivity. We then show data from additional diodes that we printed, namely a diode printed within PDMS and submerged in water, a diode printed on VHB tape and submerged in water, and the diode with yin-yang geometry.

S1. Rheological Characterization of Liquid Inks

Three different rheological tests help elucidate the behavior of a viscous liquid ink during the extrusion printing process: flow initiation, shear thinning characterization, and recovery.[1,2] In **Figure S1**, we demonstrate how the thickening agent (MC), the crosslinker (BIS), and the conductive polymer (PEDOT:PSS) influence these rheological characteristics. To provide a common point of comparison, the characterization of a reference ink composed of 12 wt.% MC, 0.7 wt.% BIS, and 1.6 wt.% PEDOT:PSS is included in every plot.

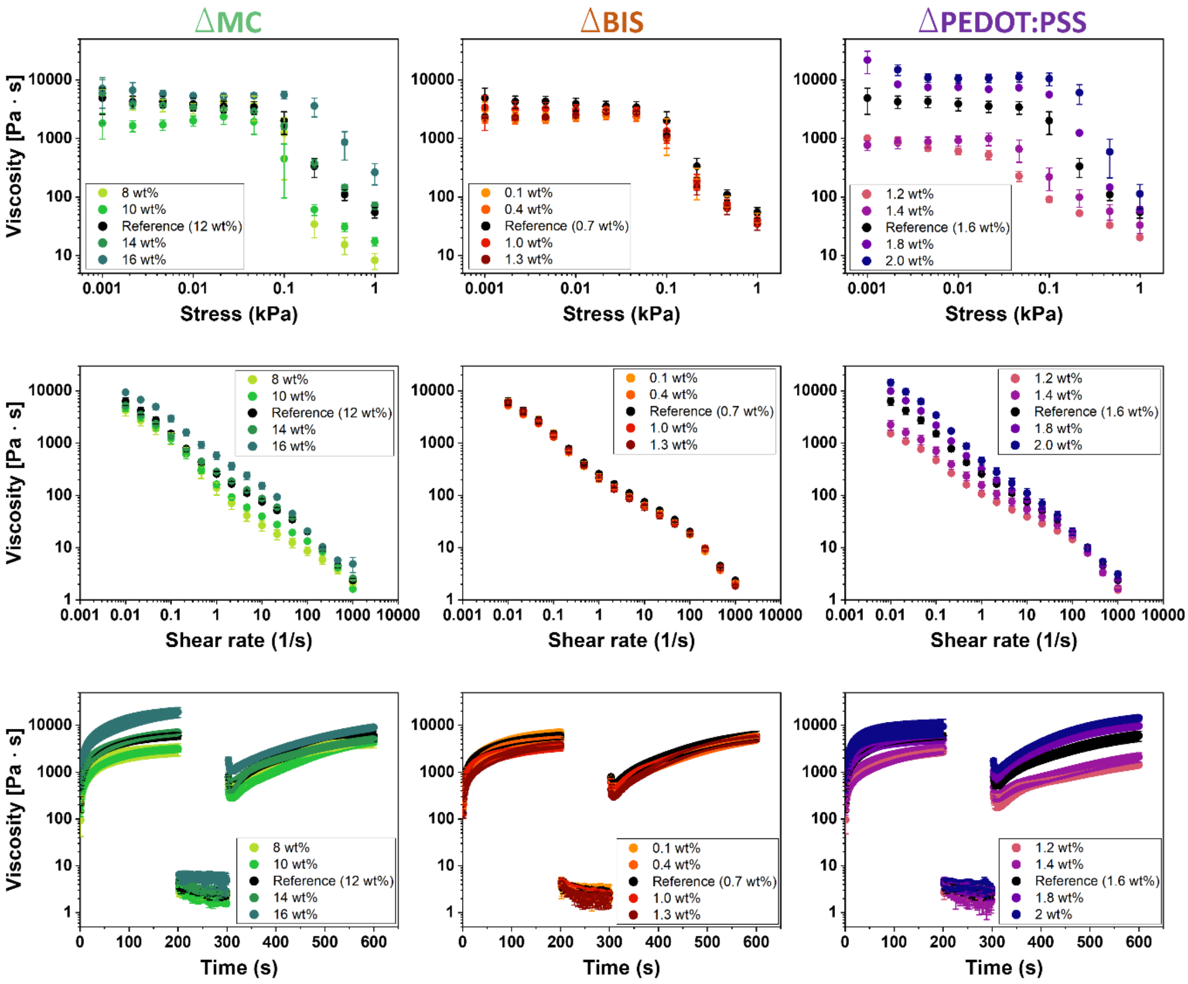

Figure S1. Rheological Characterization of fluid ink. Each column highlights the impact of one component (left: thickening agent - MC, middle: crosslinker – BIS, right: conductive polymer - PEDOT:PSS) on rheological properties by plotting five inks with different concentrations of the respective material, indicated by weight percent. Top row: The flow initiation plots reveal the yield stress at which the viscosity of each ink begins to decrease, transitioning from elastic deformation to liquid flow. Middle row: The shear thinning plots demonstrate how closely the inks behave to a Newtonian fluid and highlights the differences in viscosity at different shear rates. Bottom row: The recovery plots contain three different phases: one low shear rate phase to assess the ink in a resting state (0.01 Hz for 200 s), one high shear rate phase to simulate extrusion through the 3D printing nozzle (1000 Hz for 100 s), and another low shear rate phase (0.01 Hz for 300 s) to indicate how quickly the ink returns to its original state. One reference ink, indicated in black, is included in each plot as a consistent point of comparison. All measurements were performed in triplicate, and the results are shown as the mean with error bars that indicate the standard deviation.

	**Yield Stress [kPa]**	**Viscosity [Pa·s]**
**Reference (PEDOT:PSS 1.6 wt.%, MC 12 wt.%, BIS 0.7 wt.%)**	0.051 ± 0.005	3363 ± 577
**MC 8 wt.%**	0.041 ± 0.008	3576 ± 900
**MC 10 wt.%**	0.032 ± 0.007	2224 ± 532
**MC 14 wt.%**	0.053 ± 0.005	2780 ± 484
**MC 16 wt.%**	0.129 ± 0.014	5001 ± 231
**BIS 0.1 wt.%**	0.048 ± 0.006	2594 ± 427
**BIS 0.4 wt.%**	0.043 ± 0.002	2416 ± 322
**BIS 1.0 wt.%**	0.045 ± 0.002	2997 ± 167
**BIS 1.3 wt.%**	0.044 ± 0.002	2774 ± 133
**PEDOT:PSS 1.2 wt.%**	0.016 ± 0.001	553 ± 67
**PEDOT:PSS 1.4 wt.%**	0.025 ± 0.005	946 ± 162
**PEDOT:PSS 1.8 wt.%**	0.071 ± 0.006	5815 ± 703
**PEDOT:PSS 2.0 wt.%**	0.123 ± 0.007	9989 ± 1736

Table S1. Flow Initiation Behavior. The yield stress indicates the stress where flow is initiated and is the intersection of the fitted lines before and after flow begins. The viscosity at which the yield stress occurs is also reported. This data is derived from the plots in the top row of Figure S1 by finding the intersection of one line fitting the plateau region where the ink is deformed elastically and another line fitting the decreasing viscosity in the region indicating liquid flow. All measurements were performed in triplicate, and the results are shown as the mean ± the standard deviation.

Flow initiation indicates the yield stress at which the apparent viscosity of an ink will begin to decrease. To achieve the desired 3D printed geometry with precision, an ink should retain its shape after being printed, thus behaving somewhat like a solid rather than a fluid. As increased shear stress is applied (when being printed), the ink will reach a yield stress at which entangled polymers will become disentangled, and the ink will flow more freely.[3] Low viscosity inks that have no yield stress will flow without stress and are not ideal candidates for extrusion printing. We performed investigations to find a suitable concentration range for

each component, as inks with no yield stress would result in poor print quality and inks with a high yield stress might clog the extrusion nozzle. **Table S1** indicates the yield stress of the presented inks. The yield stress was determined using the intersection of one line fitting the plateau region where the ink is deformed elastically and another line fitting the region of flow and decreasing viscosity.[1] The PEDOT:PSS content has the greatest impact on the yield stress as well as the viscosity of the ink before flow is initiated. In contrast, the BIS content has no impact on the flow initiation behavior. The MC content seems to also have limited impact on flow initiation before a concentration threshold is reached. Four of the inks with different MC content behave quite similarly, and only the ink with the highest MC concentration demonstrates a higher yield stress.

The shear thinning characterization is used to compare the viscosity of different inks and how the inks respond to shear stresses. A perfectly Newtonian fluid will show no shear thinning or thickening behavior; the same viscosity would be retained at all shear rates.[2] The inks presented here all demonstrate shear thinning behavior, and the viscosity and degree of shear thinning can be analyzed using the power law:[1,2]

$$\eta = K\gamma^{n-1}$$

where η is the viscosity, γ is the shear rate, K indicates the viscosity at 1 s^{-1}, and n indicates the degree of shear thinning. Newtonian fluids have an n value that equals 1 whereas shear thinning fluids have a positive n value below 1, with lower values indicating a greater response to shear stress. These values are calculated for each ink in **Table S2**. Again, the BIS content shows no substantial impact on shear thinning behavior. An increasing amount of PEDOT:PSS and MC content both result in increasing viscosities. It should be noted that a similar range of viscosities are measured among the various PEDOT:PSS concentrations as the various MC concentrations, but the incremental change of PEDOT:PSS content is only 0.2 wt.% compared to the 2.0 wt.% increments used for MC content. This highlights that PEDOT:PSS has a much greater impact on viscosity than MC. Only changes in PEDOT:PSS content, though, result in noticeable changes in the degree of shear thinning. The decreasing n values indicate a further departure from classic Newtonian fluid behavior with increasing PEDOT:PSS content. Notably, the shear thinning plots are not perfectly linear, and factors that can influence this shape include the molecular weight of the components and the electrostatic interactions between components.[3] It can be expected that the charge interactions between the long PEDOT and

PSS chains will influence shear thinning behavior,[4] and MC also seems to influence molecular interactions and thus shear thinning behavior. The convergence of the shear thinning plots at high shear stresses demonstrates that the influence of electrostatic interactions will decrease with increased shear stress.[3]

	***K* [Pa·s]**	***n***
**Reference**<br>**(MC 12 wt.%, BIS 0.7 wt.%, PEDOT:PSS 1.6 wt.%)**	306 ± 14	0.39 ± 0.02
**MC 8 wt.%**	162 ± 11	0.28 ± 0.02
**MC 10 wt.%**	187 ± 15	0.37 ± 0.03
**MC 14 wt.%**	328 ± 9	0.40 ± 0.01
**MC 16 wt.%**	539 ± 23	0.35 ± 0.01
**BIS 0.1 wt.%**	278 ± 16	0.37 ± 0.02
**BIS 0.4 wt.%**	257 ± 15	0.42 ± 0.02
**BIS 1.0 wt.%**	269 ± 14	0.37 ± 0.02
**BIS 1.3 wt.%**	276 ± 16	0.33 ± 0.02
**PEDOT:PSS 1.2 wt.%**	124 ± 6	0.51 ± 0.02
**PEDOT:PSS 1.4 wt.%**	183 ± 7	0.47 ± 0.01
**PEDOT:PSS 1.8 wt.%**	369 ± 22	0.28 ± 0.01
**PEDOT:PSS 2.0 wt.%**	565 ± 22	0.27 ± 0.01

Table S2. *K* values and *n* values. The *K* value allows for the comparison of viscosity among different fluids, and would be the viscosity of a perfectly Newtonian fluid at all shear rates. The inks presented demonstrate shear thinning behavior rather than Newtonian behavior, which can be quantified with the *n* value, where n=1 indicates a Newtonian fluid and *n* values closer to 0 indicate more shear thinning behavior. This data is derived from the middle row of plots in Figure S1. All measurements were performed in triplicate, and the results are shown as the mean ± the standard deviation.

The recovery characterization demonstrates how quickly the ink returns to its original state after experiencing high stress. As discussed with the flow initiation, the ink should retain its shape after being extruded to achieve high quality prints. Although all the presented inks demonstrate relatively slow recovery,[1] the highest MC concentration, 16 wt.% MC, stands out as the ink that remains farthest from its pre-stress viscosity after 300 seconds of recovery time. Even the high concentration PEDOT:PSS inks with comparable viscosity show better recovery, indicating that high MC content beyond a threshold can result in detrimental characteristics. While this data is best visualized on the logarithmic scale of the plotted data, the percent recovery can also be used as a point of comparison (**Table S3**).

**Ink**	***Recovery at 200 s* [%]**
**Reference**	64 ± 6
**MC 8 wt.%**	115 ± 43
**MC 10 wt.%**	88 ± 21
**MC 14 wt.%**	49 ± 5
**MC 16 wt.%**	30 ± 8
**BIS 0.1 wt.%**	47 ± 12
**BIS 0.4 wt.%**	60 ± 6
**BIS 1.0 wt.%**	102 ± 21
**BIS 1.3 wt.%**	89 ± 16
**PEDOT:PSS 1.2 wt.%**	35 ± 4
**PEDOT:PSS 1.4 wt.%**	42 ± 14
**PEDOT:PSS 1.8 wt.%**	132 ± 10
**PEDOT:PSS 2.0 wt.%**	102 ± 44

Table S3. Ink Recovery. The viscosity of the ink at 200 s of low shear stress after experiencing 100 s of high shear stress is compared as a percentage to the viscosity of the ink at 200 s of low shear stress before the high stress period. Quickly recovering materials will plateau at a similar viscosity before and after the period of high shear stress. All measurements were performed in triplicate, and the results are shown as the mean ± the standard deviation.

As discussed in the Materials and Methods section of the main text, a single rheological sequence was used to characterize the material properties throughout the entire printing process, which includes: flow initiation, shear thinning, recovery, polymerization (discussed further in Section S3), and hydrogel stiffness (discussed further in Section S4). Before creating the rheological sequence, each property was measured independently. With this data, we were able to confirm that the sequence, which includes recovery periods between measurements with high shear forces, does not impact the results. These individually performed trials also confirm that the material is not dehydrating or changing in other ways throughout the measurement sequence. An example of individual measurements compared to the data gathered from the measurement sequences is provided in **Figure S2**, along with a photo of the rheological instrument.

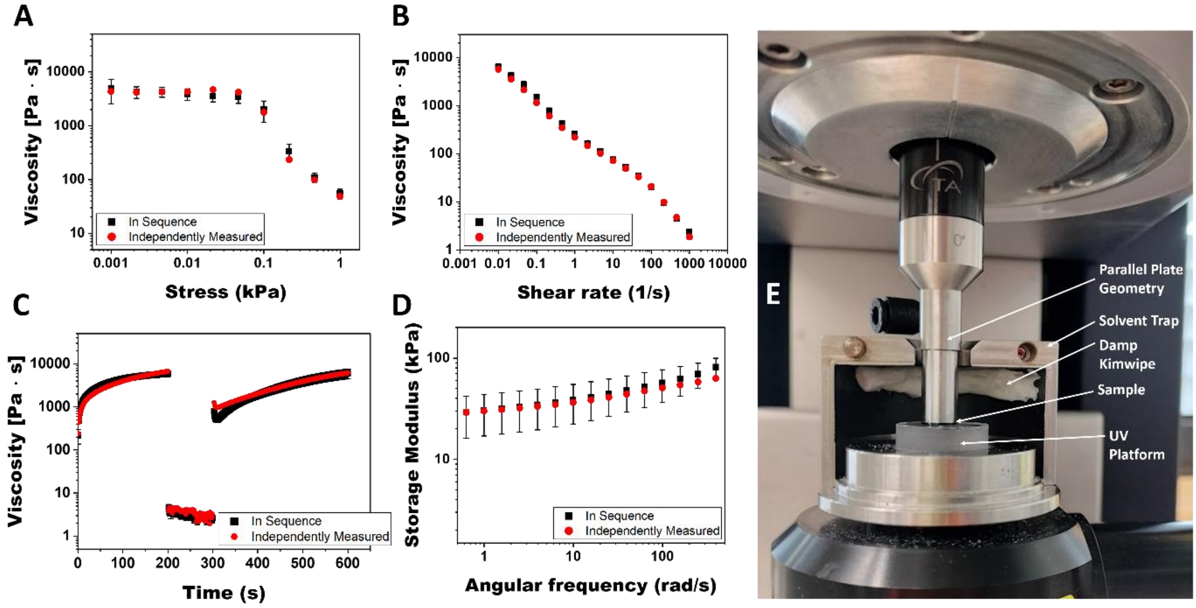

Figure S2. Validation of rheological characterization sequence. To characterize the rheological properties of the inks before, during, and after polymerization, a rheological sequence was developed. To show that the sequence had no adverse influence on the results of the measurements, a trial was conducted where each rheological test was performed independently on a separate sample. These tests include A) flow initiation, B) shear thinning, C) recovery, and D) stiffness and stability characterization after polymerization. E) A photo of the rheological instrument. The results from the sequence were performed in triplicate and are expressed as the mean of the measured values with error bars that indicate the standard deviation. The independently measured values are single measurements that corroborate the measurements from the rheological sequence.

S2. Printability Characterization

While rheological analysis can help elucidate the characteristics of inks, it does not fully encompass the useful evaluation that can come directly from extrusion print tests.[1] Although printing parameters (including nozzle diameter, print speed, and extrusion pressure) can be altered to accommodate the ink and provide the best possible print, we printed each ink with the same parameters (0.41 mm nozzle inner diameter, 20 mm/s print speed, 100 kPa pressure) to enable comparison between the different inks when printed in a dark web design, as seen in **Figure S3**. The width of a single printed line, or strut, was measured for each ink (**Table S4**) to indicate how closely the print retains the resolution of the nozzle used (0.41 mm inner diameter). Two inks, the highest concentration of PEDOT:PSS (2.0 wt.%) and of MC (16 wt.%), could not be printed using these parameters due to their high viscosity, and an extrusion pressure of 250 kPa was used instead for demonstration. Figure S3 and Table S4 reveal that the MC content has the greatest variation in print resolution under these conditions. Even though the ink with the lowest PEDOT:PSS content (1.2 wt.%) has a similar, and even slightly lower, viscosity than the ink with the lowest MC content (8 wt.%), the strut width of the 8 wt.% MC ink is much wider, indicating much more spreading during the printing process. On the other end, the highest MC content ink (16 wt.%) showed much less spreading than the highest PEDOT:PSS content ink (2.0 wt.%), even though the inks again have a similar viscosity according to Table S2. The shear thinning plots in Figure S1 demonstrate that the variation in viscosity between different inks depends on shear stress; the different PEDOT:PSS inks behave quite similarly at high shear rates compared to low shear rates. As such, the specific shear rate experienced during extrusion likely has a significant role how different concentrations of PEDOT:PSS or MC impact the printability of the ink. In this demonstration, even though the PEDOT:PSS concentration had a greater impact of the overall rheological behavior of the inks than the MC concentration, MC content ultimately had a greater impact on strut width, and thus printing resolution. Shear stress could be reduced by using less pressure or a wider nozzle, and alternatively shear stress could be increased by using greater pressure or a thinner nozzle.[5] Again, as expected based on rheological data, changes in BIS concentration had no noteworthy impact on printability. To assess the performance of each ink for printing multi-layer structures, the measurements of printed hydrogel strips are provided in **Table S7**. These strips were designed to be approximately 4

mm wide and 0.6 mm high. In future work, the extrusion nozzle, printing speed, and applied pressure could be tuned to optimize print resolution.

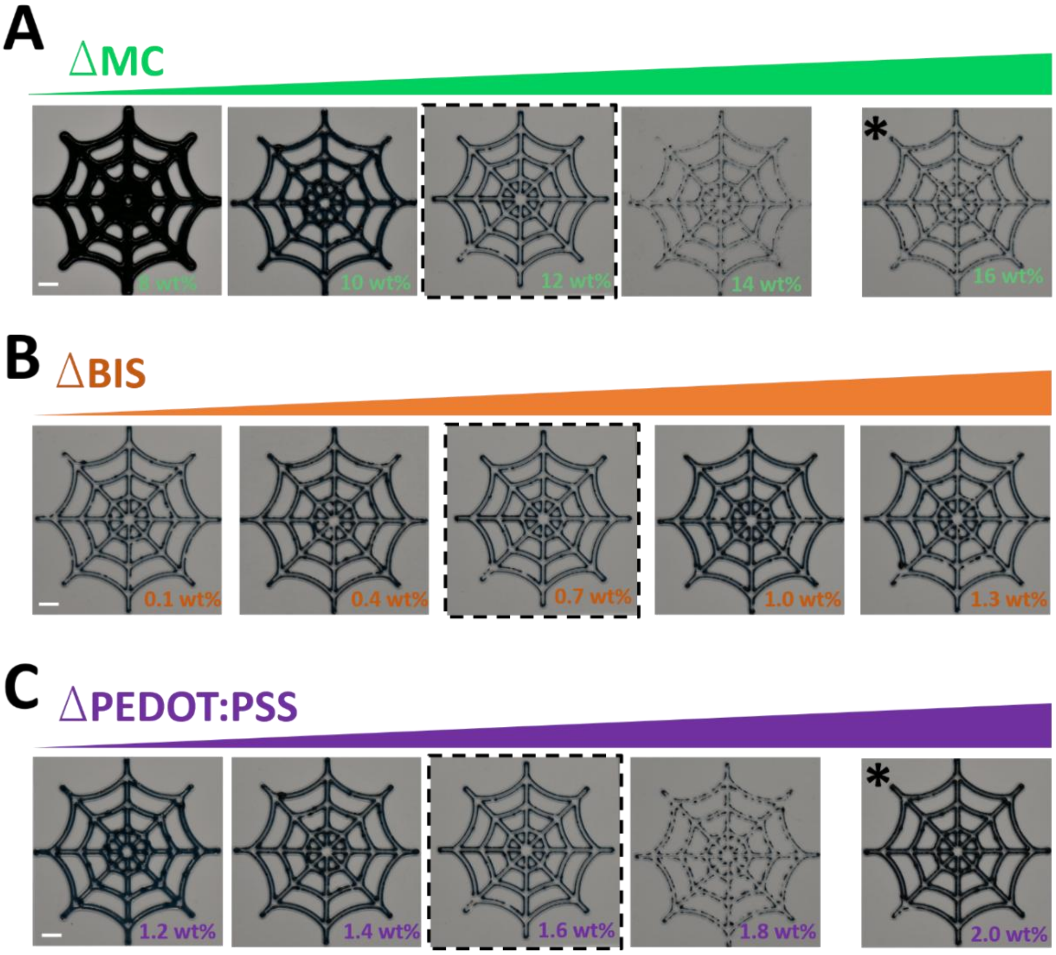

Figure S3. Ink components influence printing resolution. Inks with different concentrations of A) MC (rheological modifier), B) BIS (crosslinker), and C) PEDOT:PSS (conductive polymers) are printed using the same printing conditions (nozzle with 0.41 mm inner diameter, 100 kPa pressure, 20 mm/s speed) in a dark web design. The reference ink, shown for comparison, is framed in a black dotted frame. Marked with * in the top left corner, the highest PEDOT:PSS concentration and highest MC concentration could not be printed using the same conditions as the other inks, and instead a pressure of 250 kPa was used. All images are on the same scale and the white scale bars indicate 5 mm.

Ink	Strutt width [mm]
**Reference**	0.42 ± 0.05
**MC 8 wt.%**	1.70 ± 0.14
**MC 10 wt.%**	0.93 ± 0.08
**MC 14 wt.%**	0.37 ± 0.10
**BIS 0.1 wt.%**	0.41 ± 0.04
**BIS 0.4 wt.%**	0.41 ± 0.08
**BIS 1.0 wt.%**	0.43 ± 0.04
**BIS 1.3 wt.%**	0.42 ± 0.05
**PEDOT:PSS 1.2 wt.%**	0.68 ± 0.08
**PEDOT:PSS 1.4 wt.%**	0.57 ± 0.10
**PEDOT:PSS 1.8 wt.%**	0.35 ± 0.05

Table S4. Strut width (mm). Single lines were printed by extrusion using a nozzle with an inner diameter of 0.41 mm, a printing speed of 20 mm/s, and a pressure of 100 kPa. The strut width can indicate how well the ink retains its shape after printing. Two inks, indicated with NA, could not be printed using these conditions. Because the inks with 2.0 wt.% PEDOT:PSS and 16 wt.% MC could not be printed using the same parameters, they are excluded from this table. All measurements were performed in triplicate, and the results are shown as the mean ± the standard deviation.

S3. Characterization of Crosslinking by UV Polymerization

During the formation of the conductive hydrogel, differences in the polymerization process can influence hydrogel properties. For example, a short UV exposure would result in less crosslinking than a long UV exposure even when using the exact same ink formulation. The polymerization data shown in **Figure S4** and **Table S5** attempt to account for any potential differences that may arise due to different ink formulations. We measure the loss modulus, representative of the viscous component of the material, and the storage modulus, representative of the elastic component of the material, as the ink polymerizes due to exposure to UV irradiation beginning at the 18 second mark and lasting for 60 seconds. This process occurred on a UV platform of the rheometer while measuring the rheological properties with an 8 mm diameter flat geometry and a 0.5 mm gap between the platform and the geometry for the sample. It should be noted that these inks do not begin with the same liquid viscosity (as shown in Figure S1) nor end with the same stiffness (as discussed in the

next section) due to differences in their composition. Rather than focusing on the values of the loss modulus and storage modulus, the time at which these two values intersect can be used as an indication of polymerization time. The dark color of PEDOT:PSS can limit the penetration of UV light into the ink if the layer thickness is too great, as seen in **Figure S5**. For the 0.5 mm thickness used for rheology and the 0.2 mm height of 3D printed layers, the light-blocking effect of PEDOT:PSS on polymerization is limited.

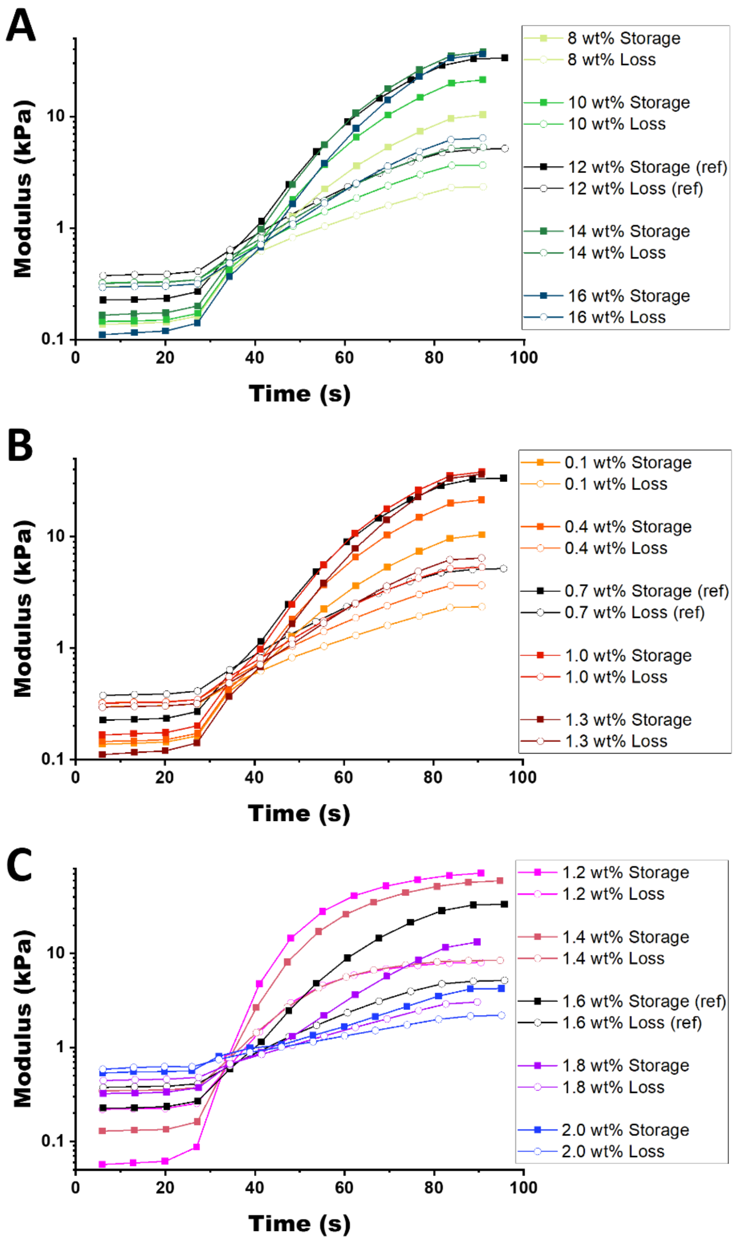

Figure S4. Analysis of UV polymerization. The loss modulus (representative of the viscous component of the material) and the storage modulus (representative of the elastic component of the material) are measured as a liquid ink is exposed to UV light, beginning at 18 seconds and lasting for 60 seconds, resulting in polymerization and the formation of a hydrogel. When the loss modulus is the dominant parameter, the material acts more like a liquid, and when the storage modulus is the dominant parameter, the material acts more like an elastic solid. The crossover point of these two parameters can be used to understand the polymerization of the material. We use representative plots to demonstrate the influence of A) MC (rheological modifier), B) BIS (crosslinker), and C) PEDOT:PSS (conductive polymers) on polymerization. Lines connecting points in all plots help guide the eye. These measurements were made in triplicate, and single representative plots of each concentration were chosen to illustrate the results to prevent overcrowding in the figure.

Ink	Time [s]
**Reference**	18.00 ± 0.29
**MC 8 wt.%**	18.47 ± 0.38
**MC 10 wt.%**	20.80 ± 1.76
**MC 14 wt.%**	17.00 ± 0.08
**MC 16 wt.%**	16.83 ± 1.22
**BIS 0.1 wt.%**	22.33 ± 1.53
**BIS 0.4 wt.%**	20.60 ± 1.16
**BIS 1.0 wt.%**	20.17 ± 1.44
**BIS 1.3 wt.%**	22.87 ± 1.03
**PEDOT:PSS 1.2 wt.%**	14.67 ± 0.04
**PEDOT:PSS 1.4 wt.%**	16.50 ± 0.08
**PEDOT:PSS 1.8 wt.%**	19.93 ± 4.00
**PEDOT:PSS 2.0 wt.%**	17.53 ± 7.85

Table S5. Time of crossover point after start of UV irradiation. The time point at which the storage modulus becomes dominant compared to the loss modulus is recorded to help assess the polymerization rate of the different formulations. All measurements were performed in triplicate, and the results are shown as the mean ± the standard deviation.

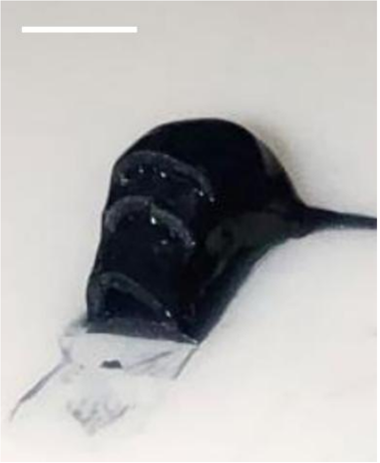

Figure S5. Photo of unpolymerized pockets when printing thick layers. If the printed layers are too thick, UV light cannot penetrate deep enough to polymerize the entire layer. The scale bar represents 5 mm.

S4. Mechanical Characterization

For solid viscoelastic materials, rheology can again be used to measure the storage modulus of the material. The storage modulus indicates the stiffness of the material, and the Young's modulus can be approximated using the storage modulus, as the Young's modulus is approximately three times larger than the storage modulus for rubber-like materials.[6] In **Figure S6 A**, we show the storage modulus for each ink at increasing angular frequencies, which demonstrates the stability of the material.[1,2,6] Reduced crosslinking is expected to reduce material stiffness, and we indeed measure a much lower storage modulus for the low BIS content hydrogel. The higher BIS content inks, however, quickly reach crosslinking saturation, and no significant difference in stiffness is measured between 0.7 wt.%, 1.0 wt.%, and 1.3 wt.% BIS hydrogels. These results suggest that, while crosslinker content is ideal for tuning hydrogel stiffness, stiffness cannot be further increased using BIS once this maximal crosslinking is reached. A trend of increasing stiffness comes from increasing MC content, with only a small effect compared to BIS and PEDOT:PSS. The PEDOT:PSS content has a very large influence on storage modulus, but the opposite trend of MC and BIS is recorded: the higher PEDOT:PSS content hydrogels have lower storage moduli. We propose that the increased content of long unaligned polymers in the double network system results in less structure and decreased stiffness.

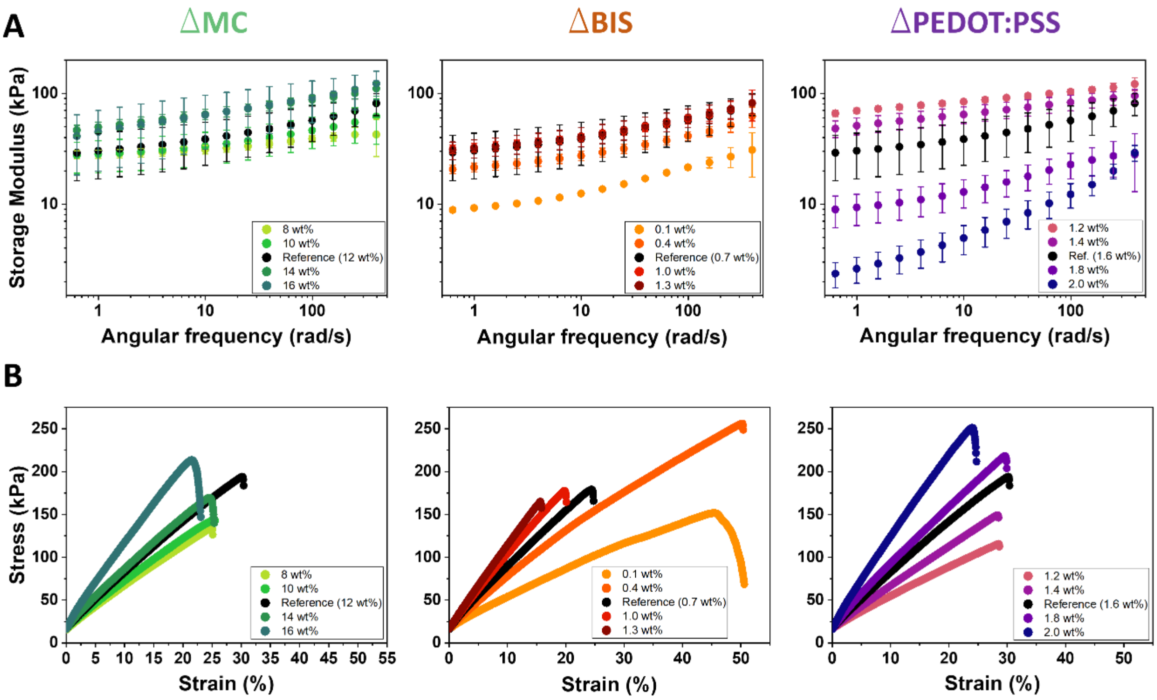

Figure S6. Material elasticity. A) Rheological analysis. The storage moduli at increasing angular frequency are shown for hydrogels with varied MC (left), BIS (middle), and PEDOT:PSS (right) content after polymerization by UV irradiation. The storage modulus can be used to approximate the Young's modulus of elastic materials, like hydrogels. One reference ink, indicated in black, is included in each plot as a consistent point of comparison. All measurements were performed in triplicate, and the results are shown as the mean with error bars that indicate the standard deviation. B) Tensile characterization. Representative tensile characterization plots of hydrogel strips (approximately 10 mm wide, 0.6 mm thick, and 45 mm long) that were 3D printed using inks of varying MC (left), BIS (middle), and PEDOT:PSS (right) content. One reference ink, indicated in black, is included in each plot as a consistent point of comparison. These trials were performed in triplicate, and these plots show single representative measurements of each formulation to avoid overcrowding the data.

Elasticity can additionally be assessed using tensile characterization. The slope of the stress-strain plot can also indicate Young's modulus, the shape of the plot can reveal material behavior, and the material's maximum strain can be indicated by the breaking point.[7] We 3D printed a band (dimensions: width – 10 mm, thickness – 0.6 mm, length – 30 mm) of each hydrogel material to assess the tensile behavior (**Figure S6 B**). The various BIS and MC hydrogels demonstrate similar trends as revealed with during the rheological assessment. The 0.1 wt.% BIS hydrogel demonstrates the most gradual slope, stretching the farthest with the

least amount of force. Relatedly, the lowest BIS content hydrogels achieve the highest strain before breaking. As maximal crosslinking is approached with the reference hydrogel (0.7 wt.% BIS), increased BIS content results in only minor change to tensile behavior. Changes in MC content has relatively little impact on tensile behavior, with the highest MC content hydrogel (16 wt.%) being the outlier. The different PEDOT:PSS hydrogels again show a clear trend in stiffness, however, surprisingly, the trend is the opposite of that found with rheology. Whereas rheology indicated that the increasing PEDOT:PSS resulted in decreased stiffness, tensile tests indicate increasing stiffness with increasing PEDOT:PSS content. We propose this finding indicates that the PEDOT:PSS fibers become aligned during the extrusion printing process, an effect that has been previously observed from extrusion printed PEDOT:PSS.[4] For the rheological assessment, the hydrogel is essentially in a cast mold, sandwiched between the platform and the rotational plate, when it is polymerized. The PEDOT:PSS polymer networks can have a random alignment in this method, and are not likely to be extended. During extrusion printing, the shear forces likely align the fiber network. Previous work indicates that increased shear forces result in increased alignment.[4,8] A few examples of factors that impact shear force include extrusion pressure, nozzle diameter, and ink viscosity, which could be increased with the use of a higher PEDOT:PSS concentration, for example. Using extrusion 3D printing, the aligned polymers are already somewhat elongated, limiting the material's ability to stretch. Although it could have been expected that the rheology and tensile characterizations would reveal the same trends, as they did with BIS and MC, the reversed trend discovered with PEDOT:PSS highlights how the fabrication method can influence material properties.

S5. Conductivity Characterization

We used multiple techniques to evaluate the conductivity of the conductive hydrogels (**Figure S7**). For one set of measurements, graphite felt electrodes soaked with a hexacyanoferrate (HCF) solution as a redox agent to accommodate ionic conductivity.[9] A separate set of measurements were made using traditional platinum electrodes and a 4-point probe method, demonstrating that this material can interface with both electronic and iontronic systems. As expected, increasing the conductive polymer (PEDOT:PSS) content results in increased conductivity. However, increased MC content does result in decreased conductivity. Meanwhile, the crosslinker (BIS) content seems to have no impact on

conductivity. This finding highlights another advantage of utilizing conductive polymers; hydrogels that use ionic atoms, like common salts, as charge carries will have reduced conductivity with increased crosslinker concentration because the diffusion path of the ions becomes more convoluted.[10] The conductive pathways provided by the conductive polymers presented here seem to be unaffected by crosslinking density at the concentrations investigated. Additionally, the interpenetrated network of conductive polymers will not diffuse out of the hydrogel when submerged in water like atomic ions would. This allows these hydrogels to be used in aqueous environments, and thus we also measured conductivity of the material after it was soaked in deionized water for one hour. The hydrated gels always exhibited lower conductivity largely due to the swelling that occurs. Because the cross-sectional area was used in calculating conductivity, we can also observe how each component influenced swelling (**Table S7**). To provide deeper understanding, future work could model hydrogel swelling, using the Flory-Rehner equation for example, and the impact on ion flow.[11–13]

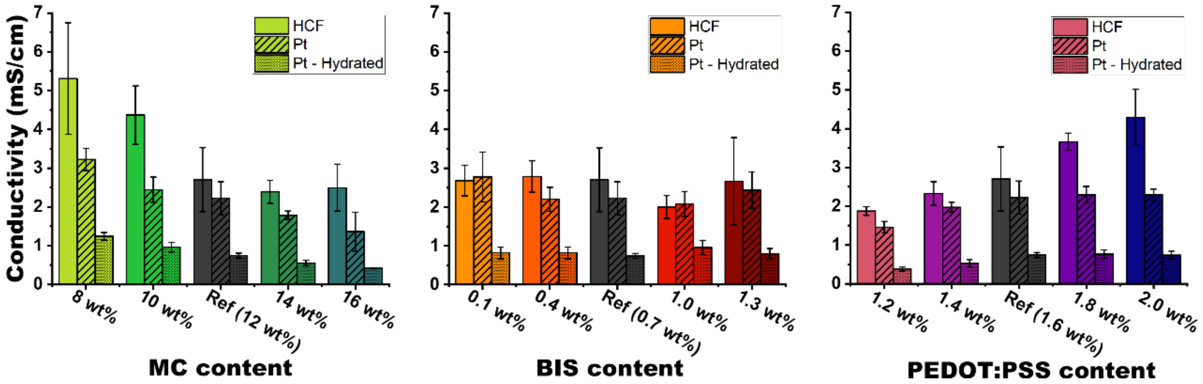

Figure S7. Conductivity Characterization. Conductivity measurements performed on 3D printed samples using inks with varying MC (left), BIS (middle), and PEDOT:PSS (right) content. One set of measurements was taken using graphite felt electrodes soaked with and HCF solution (HCF). The other measurements were taken using the 4-point probe method using platinum electrodes (Pt). The hydrated (Pt-Hydrated) sample was measured after the 3D printed material soaked in deionized water for one hour. Other measurements were made immediately after printing. One reference ink, indicated in black, is included in each plot as a consistent point of comparison. All measurements were performed in quadruplicate, and the results are shown as the mean with error bars that indicate the standard deviation.

Ink	Printed Width [mm]	Printed Height [mm]	Swollen Width [mm]	Swollen Height [mm]	Swollen area [%]
**Reference**	4.2 ± 0.1	0.6 ± 0.1	5.2 ± 0.2	0.9 ± 0.2	190 ± 50
**MC 8 wt.%**	4.9 ± 0.2	0.8 ± 0.1	5.7 ± 0.3	0.9 ± 0.1	130 ± 20
**MC 10 wt.%**	4.6 ± 0.2	0.7 ± 0.1	5.3 ± 0.4	0.8 ± 0.1	130 ± 30
**MC 14 wt.%**	4.6 ± 0.2	0.8 ± 0.1	5.9 ± 0.3	1.0 ± 0.1	160 ± 30
**MC 16 wt.%**	3.8 ± 0.1	0.4 ± 0.1	5.2 ± 0.2	0.7 ± 0.1	240 ± 70
**BIS 0.1 wt.%**	3.7 ± 0.1	0.5 ± 0.1	5.3 ± 0.3	0.9 ± 0.1	260 ± 60
**BIS 0.4 wt.%**	3.9 ± 0.1	0.5 ± 0.1	5.3 ± 0.3	0.9 ± 0.1	240 ± 60
**BIS 1.0 wt.%**	4.4 ± 0.1	0.6 ± 0.1	4.9 ± 0.1	0.9 ± 0.1	170 ± 30
**BIS 1.3 wt.%**	4.4 ± 0.2	0.7 ± 0.1	5.5 ± 0.5	0.9 ± 0.1	160 ± 30
**PEDOT:PSS 1.2 wt.%**	3.9 ± 0.1	0.5 ± 0.1	5.8 ± 0.1	1.0 ± 0.1	300 ± 70
**PEDOT:PSS 1.4 wt.%**	3.9 ± 0.1	0.7 ± 0.1	5.4 ± 0.1	1.0 ± 0.1	200 ± 40
**PEDOT:PSS 1.8 wt.%**	4.3 ± 0.1	0.6 ± 0.1	5.5 ± 0.1	0.9 ± 0.1	190 ± 40
**PEDOT:PSS 2.0 wt.%**	4.2 ± 0.2	0.5 ± 0.1	5.7 ± 0.1	0.8 ± 0.1	220 ± 50

Table S7. Swelling characterized by cross-sectional area. The width and height of 3D printed hydrogel strips were measured before and after being submerged in distilled water for approximately 1 hour. The target dimensions for the printed structure were a width of 4 mm and a height of 0.6 mm. The cross-sectional area was used to calculate conductivity in different conditions (Figure S7). The relative cross-sectional area of the swollen hydrogel is shown as a percentage of the cross-sectional area before soaking. All measurements were performed in quadruplicate, and the results are shown as the mean ± the standard deviation.

S6. Diode Variations

The chemicals used to functionalize the three different hydrogels used to create ionic diodes are shown in **Figure S8** to help demonstrate underlying mechanisms. Within PEDOT:PSS, the PEDOT chain facilitates the transfer of electrons whereas the PSS chain facilitates the transfer of ions. The acrylate groups on 2-Acrylamido-2-methylpropane sulfonic acid (AMPS) and (3-acrylamidopropyl) trimethylammonium (APTAC) allow for negatively and positively charged groups, respectively, to be covalently incorporated into the acrylamide hydrogel network. In the main work, hydrogels functionalized with AMPS have been shown

as green and the hydrogels functionalized with APTAC have been shown as yellow to help provide clear demonstrations. When these gels are not dyed, they are in fact transparent, as seen in Figure S8.

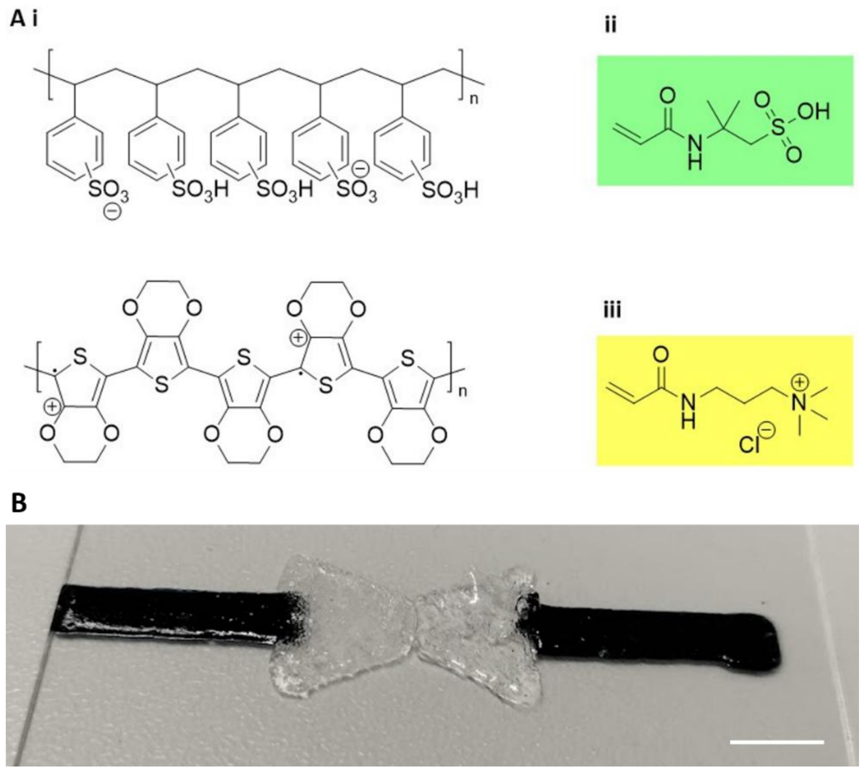

Figure S8. The components of ionic diodes. A) The chemical structures of i) PEDOT:PSS, ii) AMPS, and iii) APTAC. B) An image of the ionic diode printed on a glass surface without added color. The scale bar represents 10 mm.

We tested the ionic diode in a variety of conditions to evaluate the robustness of this implementation. Notably, this version of the ionic diode could remain stable when exposed to deionized water, as seen in **Figure S9 A**. Previous demonstrations that used atomic ions as charge carriers would not maintain functionality when the ions are able to diffuse out of the ionic diode into a larger bath solution. In this figure, we also show the forward and reverse voltage scan. Current rectification is best shown in the forward direction; the peak shown in the reverse direction is a function of discharge time and is reduced at slow scanning speeds.[14,15] We show here that we observe the expected shape when scanning in both directions, and we otherwise focus on the forward voltage scan for the diodes demonstrated in this work.

We also wanted to show that the ionic diode could be printed on substrates other than the PDMS ink developed in this work. Previous work fabricated elastic ionic diodes using VHB tape,[16,17] so we showed that we can also print the functionalized hydrogels onto VHB tape. This substrate is much more elastic than the PDMS ink. After sandwiching the printed diode under another layer of VHB tape, the diode remained functional after being folded in half. This was then submerged in water, and again the diode remained stable (**Figure S9 B**).

While the ionic diodes shown in the main text have rectangular and trapezoidal shapes, organic shapes can also be printed to fabricate functional ionic diodes. In **Figure S9 C**, we show the current rectification of a yin-yang shaped diode. Because this geometry has no thin sections, the current flow when a positive voltage is applied is high in comparison to many of the other diodes demonstrated in this work.

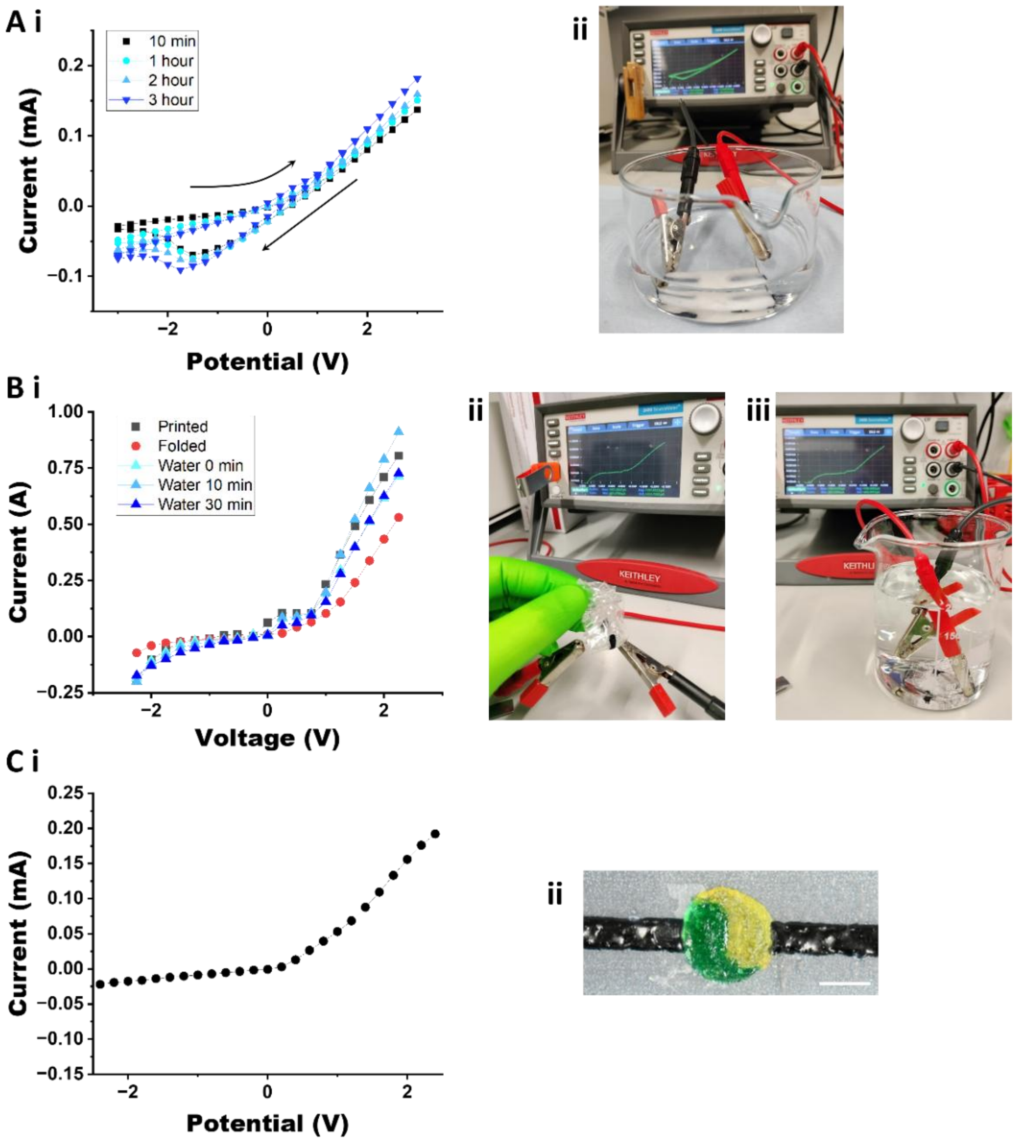

Figure S9. Exploration into the robustness of the 3D printed ionic diode. A) The ionic diode maintains functionality even when exposed and submerged in water for extended periods of time. i) The IV plot of an ionic diode with a trapezoid geometry in water at different times. In this plot, the forward voltage scan and reverse voltage scan is shown, as indicated by arrows. In all other IV plots, just the forward voltage scan is shown. ii) A photo of the diode submerged in deionized water. The PEDOT:PSS functionalized hydrogels are exposed on the ends where they connect to silver chloride electrodes while the rest of the diode is encased in PDMS. B) An ionic diode printed on a VHB tape substrate is folded and submerged in water. i) The IV plot of the ionic diode on a VHB tape substrate after being printed, after being folded, and after being submerged in water. ii) An image of the folded diode. iii) An image of the folded diode submerged in deionized water. C) An ionic diode with a yin-yang geometry. i) The IV plot of the yin-yang diode. ii) An image of the yin-yang diode dyed following the scheme of the main text for demonstration purposes only. Measurements were taken using a sample without dye added. The scale bar indicates 5 mm. The lines in all plots are only to help guide the eye.

References

[1] N. Paxton, W. Smolan, T. Böck, F. Melchels, J. Groll, T. Jungst, Proposal to assess printability of bioinks for extrusion-based bioprinting and evaluation of rheological properties governing bioprintability, Biofabrication 9 (2017) 044107. https://doi.org/10.1088/1758-5090/aa8dd8.

[2] E.A. Kiyotake, A.W. Douglas, E.E. Thomas, S.L. Nimmo, M.S. Detamore, Development and quantitative characterization of the precursor rheology of hyaluronic acid hydrogels for bioprinting, Acta Biomater. 95 (2019) 176–187. https://doi.org/10.1016/j.actbio.2019.01.041.

[3] J.L. Dávila, M.A. d'Ávila, Laponite as a rheology modifier of alginate solutions: Physical gelation and aging evolution, Carbohydr. Polym. 157 (2017) 1–8. https://doi.org/10.1016/j.carbpol.2016.09.057.

[4] H. Yuk, B. Lu, S. Lin, K. Qu, J. Xu, J. Luo, X. Zhao, 3D printing of conducting polymers, Nat. Commun. 11 (2020) 1604. https://doi.org/10.1038/s41467-020-15316-7.

[5] R. Chand, B.S. Muhire, S. Vijayavenkataraman, Computational Fluid Dynamics Assessment of the Effect of Bioprinting Parameters in Extrusion Bioprinting, Int. J. Bioprinting 8 (2022) 545. https://doi.org/10.18063/ijb.v8i2.545.

[6] T.K.L. Meyvis, B.G. Stubbe, M.J. Van Steenbergen, W.E. Hennink, S.C. De Smedt, J. Demeester, A comparison between the use of dynamic mechanical analysis and oscillatory shear rheometry for the characterisation of hydrogels, Int. J. Pharm. 244 (2002) 163–168. https://doi.org/10.1016/S0378-5173(02)00328-9.

[7] J.L. Drury, R.G. Dennis, D.J. Mooney, The tensile properties of alginate hydrogels, Biomaterials 25 (2004) 3187–3199. https://doi.org/10.1016/j.biomaterials.2003.10.002.

[8] A. Sydney Gladman, E.A. Matsumoto, R.G. Nuzzo, L. Mahadevan, J.A. Lewis, Biomimetic 4D printing, Nat. Mater. 15 (2016) 413–418. https://doi.org/10.1038/nmat4544.

[9] A. Guha, T.J. Kalkus, T.B.H. Schroeder, O.G. Willis, C. Rader, A. Ianiro, M. Mayer, Powering Electronic Devices from Salt Gradients in AA-Battery-Sized Stacks of Hydrogel-Infused Paper, Adv. Mater. 33 (2021) 2101757. https://doi.org/10.1002/adma.202101757.

[10] Y. Wu, S. Joseph, N.R. Aluru, Effect of Cross-Linking on the Diffusion of Water, Ions, and Small Molecules in Hydrogels, J. Phys. Chem. B 113 (2009) 3512–3520. https://doi.org/10.1021/jp808145x.

[11] R.G.M. van der Sman, Biopolymer gel swelling analysed with scaling laws and Flory–Rehner theory, Food Hydrocoll. 48 (2015) 94–101. https://doi.org/10.1016/j.foodhyd.2015.01.025.

[12] A. Erdem, F.A. Ngwabebhoh, U. Yildiz, Synthesis, characterization and swelling investigations of novel polyetheramine-based hydrogels, Polym. Bull. 74 (2017) 873–893. https://doi.org/10.1007/s00289-016-1751-y.

[13] N. Ramasamy, A. Sivalingam, S.S. Saravanabhavan, K.N. Palani, B. Natesan, Preparation and evaluation of physicochemical studies of novel natural cellulose microfibril (CMF) reinforced poly (sodium acrylate) hydrogel, Carbon Lett. 34 (2024) 743–755. https://doi.org/10.1007/s42823-023-00592-2.

[14] J.-H. Han, K.B. Kim, J.H. Bae, B.J. Kim, C.M. Kang, H.C. Kim, T.D. Chung, Ion Flow Crossing Over a Polyelectrolyte Diode on a Microfluidic Chip, Small 7 (2011) 2629–2639. https://doi.org/10.1002/smll.201100827.

[15] S.H. Han, S.I. Kim, H.-R. Lee, S.-M. Lim, S.Y. Yeon, M.-A. Oh, S. Lee, J.-Y. Sun, Y.-C. Joo, T.D. Chung, Hydrogel-Based Iontronics on a Polydimethylsiloxane Microchip, ACS Appl. Mater. Interfaces 13 (2021) 6606–6614. https://doi.org/10.1021/acsami.0c19892.
[16] H.-R. Lee, J. Woo, S.H. Han, S.-M. Lim, S. Lim, Y.-W. Kang, W.J. Song, J.-M. Park, T.D. Chung, Y.-C. Joo, J.-Y. Sun, A Stretchable Ionic Diode from Copolyelectrolyte Hydrogels with Methacrylated Polysaccharides, Adv. Funct. Mater. 29 (2019) 1806909. https://doi.org/10.1002/adfm.201806909.
[17] Y. Wang, Z. Wang, Z. Su, S. Cai, Stretchable and transparent ionic diode and logic gates, Extreme Mech. Lett. 28 (2019) 81–86. https://doi.org/10.1016/j.eml.2019.03.001.
